# Encephalomyocarditis virus protein 2B* interacts with 14-3-3 proteins through a phosphorylated C-terminal binding motif

**DOI:** 10.1128/mbio.01008-25

**Published:** 2025-08-18

**Authors:** Samantha K. Nguyen, Nadine Anders, Stephen Holmes, Henry G. Barrow, Nina Lukhovitskaya, Aminu S. Jahun, Iliana Georgana, Laura G. Caller, James R. Edgar, Stephen C. Graham, Edward Emmott, Andrew E. Firth, Hazel Stewart

**Affiliations:** 1 Department of Pathology, University of Cambridge 98523, Cambridge, United Kingdom; 2 Department of Pharmacology, University of Cambridge 85444, Cambridge, United Kingdom; 3 Centre for Proteome Research, Department of Biochemistry, Cell & Systems Biology, Institute of Systems, Molecular and Integrative Biology, University of Liverpool 415009 , Liverpool, United Kingdom; Duke University School of Medicine, Durham, North Carolina, USA; Stanford University School of Medicine, Stanford, California, USA

**Keywords:** picornavirus, EMCV, interferon beta, cardiovirus, ribosome frameshifting, innate immunity, proteomics, 14-3-3 proteins, 2B*

## Abstract

Encephalomyocarditis virus (EMCV) has been an important model RNA virus for decades. Although most of the EMCV proteins are obtained via proteolytic cleavage of a long polyprotein, 2B* is expressed from a short overlapping open reading frame via an unusual protein-stimulated temporally dependent ribosomal frameshifting mechanism. Mutant viruses that are unable to express 2B* have a small plaque phenotype due to delayed onset and inefficient progression of multiple lytic cell death pathways. However, the mechanism by which 2B* promotes these pathways has not yet been characterized. By tagging 2B* within the viral genome, we identified putative interaction partners of 2B* and showed that 2B* binds all seven members of the 14-3-3 protein family during virus infection. Binding is entirely dependent on a mode III motif containing a phosphoserine (RRNpSS) at the 2B* C terminus. This may impede other functions of the 14-3-3 proteins, including their role in promoting antiviral signaling. Ablating the 2B*:14-3-3 interaction had no effect upon plaque size, indicating that the function of this interaction is unrelated to the role of 2B* in promoting lytic cell death. This work expands our knowledge of the protein complement of this important model virus and the binding repertoire and specificity of host 14-3-3 proteins.

**IMPORTANCE:**

Encephalomyocarditis virus (EMCV) infects a range of species, causing economically important reproductive disorders in pigs and encephalitis and myocarditis in rodents. Due to its wide host range, it is an important model pathogen for investigating virus-host interactions. EMCV expresses an accessory protein, 2B*, from an overlapping open reading frame via an unusual ribosomal frameshifting mechanism. Although the frameshifting mechanism has been established, the function of the 2B* protein had not been explored until recently. Here, we determined the host proteins to which 2B* binds and found that it specifically binds to all members of the 14-3-3 protein family, which, among other roles, contribute to the innate immune response to viral infection in mammalian cells. The interaction requires a specific stretch of amino acids at the end of 2B*. Binding to 2B* may reduce the opportunities for these 14-3-3 proteins to bind to host proteins and perform their usual roles; therefore, by interacting with the 14-3-3 proteins, 2B* may affect multiple host cell functions, including immune response activation.

## INTRODUCTION

Encephalomyocarditis virus (EMCV) is a positive-sense, single-stranded RNA virus in the genus *Cardiovirus* of the family *Picornaviridae*. EMCV causes encephalitis and myocarditis in a variety of species, as well as reproductive disorders in pigs. The genome is approximately 8 kb in length and encodes a long polyprotein, which is cleaved to produce 12 mature virus proteins. Although most of the polyprotein processing is performed by the viral 3C protease, separation between 2A and 2B occurs via the StopGo mechanism, whereby a specific amino acid sequence ending in NPGP co-translationally prevents the formation of a peptide bond between the G and the final P (1). Just 12 codons downstream of the 2A|2B junction lies a programmed ribosomal frameshifting (PRF) site at which a proportion of ribosomes are stimulated to make a −1 nt shift into the 117-codon overlapping 2B* open reading frame (ORF) (2). The shift site comprises a G GUU UUU heptanucleotide (spaces separate polyprotein-frame codons), and the stimulator comprises a 3′ RNA structure that binds the viral 2A protein to form an RNA:protein complex that impedes ribosome processivity (3, 4). PRF leads to the production of the 129 amino acid “transframe” protein 2B* and prevents translation of the downstream nonstructural proteins. The efficiency of PRF is regulated by the increasing concentration of 2A over time, with the percentage of frameshifting ribosomes increasing from ~0% at 2 h post-infection (hpi) to ~70% at 6–8 hpi (3). Thus, both the expression level of 2B* and the ratio of structural to nonstructural proteins are temporally controlled.

Although 2B* is highly conserved between EMCV isolates, it is not encoded by other picornaviruses, even the closely related cardiovirus, Theiler’s murine encephalomyelitis virus (TMEV). PRF does occur at the same site in TMEV, but it leads to the expression of a peptide only 14 residues in length that has no known function (5). Therefore, in TMEV, it is thought that PRF is used purely as a “ribosome sink” to temporally control the ratio of structural to nonstructural protein synthesis (5, 6). 2B* therefore represents an uncharacterized protein, present in a subgroup of cardioviruses. The loss of 2B* in EMCV has been associated with reduced viral plaque size (2, 3), and we recently showed that this is due to decreased rates of lytic virus release mediated by two distinct programmed cell death pathways, both of which are enhanced by the presence of 2B* through an as-yet unidentified mechanism (7).

Here, we modified the EMCV genome to encode an HA-tagged 2B* protein. Using this virus, we identified the host protein binding partners of 2B* and found that they include the entire family of 14-3-3 proteins. A short linear motif at the C terminus of 2B* is essential for 14-3-3 binding, forming a mode III interaction site. However, this interaction does not contribute to the previously described role of 2B* in promoting plaque size (7), but instead leads to reduced transcription of both *IFNB1* and *IL6*. Although a detailed molecular mechanism for this was not established, it is consistent with recently published results describing a role of 14-3-3 proteins in promoting antiviral immunity (8–11). 2B* may possess unknown additional functions reliant on this 14-3-3 sequestration.

## RESULTS

### Identification of a 2B*KO EMCV mutation that does not affect viral frameshifting or replication

As the 2B* ORF overlaps the coding sequence of 2B, we first sought to confirm that mutations applied to create a 2B* knock-out (2B*KO) virus do not affect either viral PRF efficiency or RNA replication. Several 2B*KO EMCV mutants have been described previously (2, 12), one of which possesses two stop codons immediately after the RNA stem-loop, which are synonymous in the 2B reading frame (WT-PTC mutant of reference 12). In this virus, PRF results in the expression of a severely truncated, 29-residue N-terminal fragment of the 129-residue 2B*.

**Fig 1:**
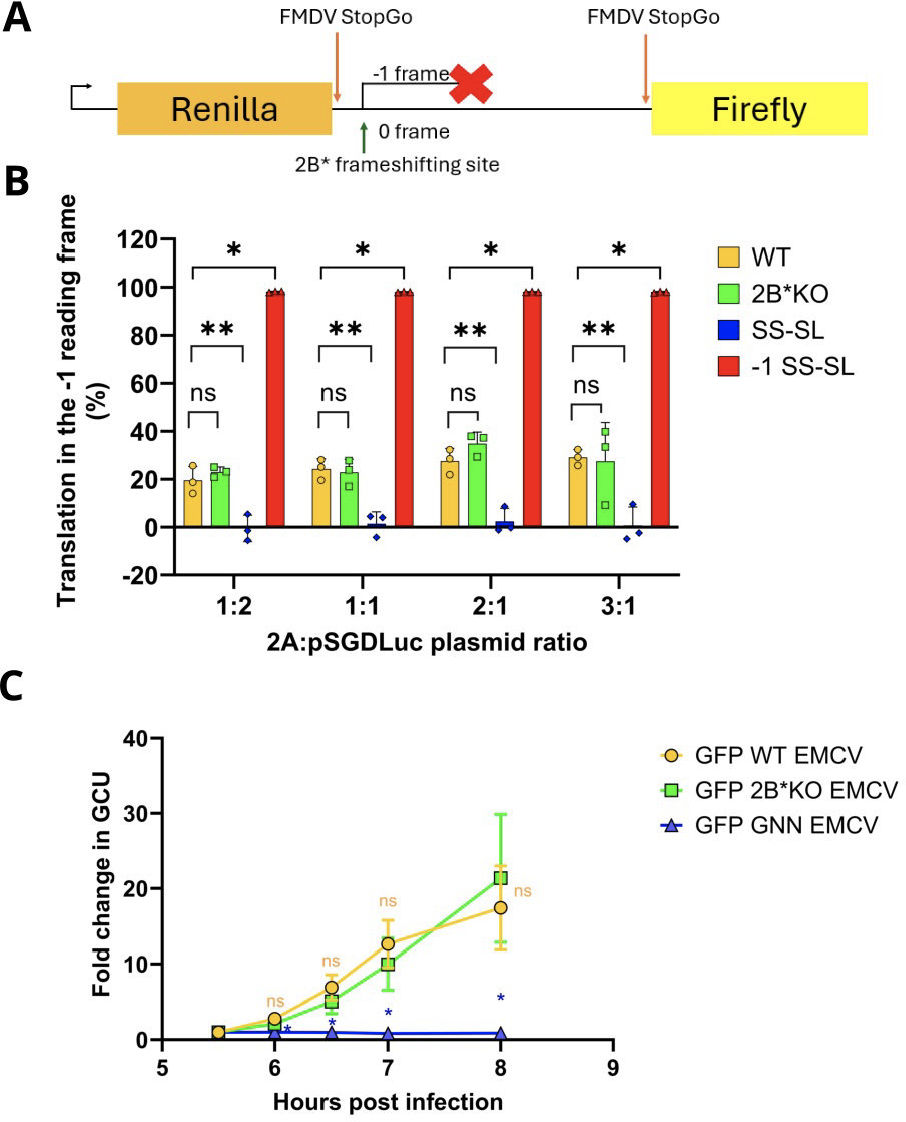
2B*KO EMCV mutations do not impact viral frameshifting or replication. (**A**) Schematic representation of the dual luciferase constructs. Renilla luciferase is produced by every translating ribosome. However, in the WT sequence, firefly luciferase is only produced when frameshifting does not occur. This enables quantitative measurement of the percentage of ribosomes in each reading frame for each mutant construct. (**B**) BHK-21 cells were transiently co-transfected with a WT EMCV 2A expression construct (pCAGG-2A) along with a dual luciferase plasmid containing 105 nt of the EMCV genome (WT pSGDLuc) or mutants thereof (2B*KO pSGDLuc, SS-SL pSGDLuc, or −1 SS-SL pSGDLuc) at various ratios. The SS-SL cassette contains mutations in both the slippery sequence and the stem-loop, which collectively ablate PRF. The −1 SS-SL cassette contains the SS-SL mutations, as well as an additional single nucleotide insertion, ensuring all ribosomes enter the second ORF. This construct therefore mimics 100% frameshifting. The 2B*KO cassette contains the premature termination codons, used elsewhere in this manuscript, designed to ablate 2B* expression while leaving PRF rates unaffected. At 24 h post-transfection, cells were frozen in 1× passive lysis buffer, and both renilla and firefly luciferase activities were measured. Samples were normalized to the luciferase values for the same pSGDLuc construct co-transfected with pCAGG-2Amut. The percentages of ribosomes in the 0 or −1 reading frames for each ratio of pCAGG-2A:pSGDLuc were calculated. Data shown are the mean ± SD of three biological repeats, each using triplicate wells. Statistical analysis (Student’s *t*-test): ns, not significant; *P ≤ 0.05; and *P ≤ 0.01. (**C**) A confluent monolayer of BHK-21 cells was infected with GFP WT EMCV or GFP 2B*KO EMCV at an MOI of 0.01. An equivalent volume of cell lysate from GFP GNN EMCV-transfected cells was also included as an additional control. GCU was analyzed using the Incucyte live-cell imaging suite (Sartorius) and normalized to the respective GCU at 5.5 hpi. Data shown are the mean ± SD of three biological repeats, each using triplicate wells. Statistical analysis (Student’s *t*-test of the indicated virus compared to GFP WT EMCV): ns, not significant; *P ≤ 0.05.

As PRF in EMCV directs ribosomes out of the polyprotein ORF into the 2B* ORF, any effect of the 2B*KO mutation on PRF efficiency would affect the ratio of structural to nonstructural protein synthesis at late stages of infection, potentially overshadowing any phenotype caused by the loss of 2B*. While previous work with a metabolic labeling assay (12) indicated that the same 2B*KO mutation did not have a measurable effect on PRF, we sought to confirm this with an alternative, dual luciferase-based assay (Fig. 1A and B).

For each sequence tested, the shift site, stem-loop, and any downstream mutations were inserted into the previously described pSGDLuc plasmid (Fig. 1A) (13). The resulting plasmids contained 11 nt upstream of the slippery sequence, the 7 nt slippery sequence itself, and an additional downstream 87 nt, between renilla and firefly luciferase ORFs, which were in the same reading frame (0 frame) (Fig. 1A). Flanking the inserted nucleotides were two FMDV StopGo sequences, enabling co-translational separation, to ensure that the peptide sequences encoded by the insert are not tagged onto the end of the renilla and/or firefly luciferases where they might differentially affect enzymatic activities. Ribosomes that do not frameshift translate both luciferase proteins, whereas ribosomes that frameshift translate only the renilla luciferase (Fig. 1A).

As binding of the EMCV 2A protein to the stem-loop is necessary for PRF, a construct encoding FLAG-tagged EMCV 2A (pCAGG-2A) was co-transfected with each pSGDLuc construct. To allow normalization of the relative luciferase readings, each pSGDLuc construct was also co-transfected with a plasmid encoding a FLAG-tagged 2A mutant (pCAGG-2Amut) that is unable to bind the stem-loop and unable to stimulate PRF (3). The PRF efficiency could then be calculated as 1 − (FLuc_test_/RLuc_test_)/(FLuc_2Amut_/RLuc_2Amut_). As the ratio of pCAGG-2A to pSGDLuc, which would mimic the ratio of 2A protein to viral RNA occurring in infection, was unknown, a range of co-transfection ratios was tested (Fig. 1B).

A previously described mutant sequence, non-permissive to PRF due to mutations in both the slippery sequence and stem-loop (SS-SL) (3), was also inserted in the pSGDLuc vector as an additional control. A further single-nucleotide insertion was added to this construct to create −1 SS-SL. Here, the SS-SL mutations prevented PRF, and the insertion caused all ribosomes to enter the 2B*-derived reading frame, thus mimicking 100% PRF. As expected, pSGDLuc-2B*KO showed no significant difference in PRF efficiency compared to pSGDLuc-WT, indicating that the 2B*KO mutations do not affect PRF, and therefore, any phenotypic differences found between 2B*KO EMCV and WT EMCV in later experiments would not be due to impaired PRF efficiency.

In principle, any change to the viral RNA genome may also influence viral RNA replication. 2B* mutant viruses have previously been observed to reach equivalent overall titers to WT (2, 7), despite the small plaque phenotype. Furthermore, 2B* is only expressed at late time points, after substantial amounts of RNA replication have already occurred. Therefore, any difference in viral replication rates between 2B*KO EMCV and WT EMCV would likely be due to changes in RNA sequence or RNA structure, rather than a direct function of the 2B* protein. To further validate the use of 2B*KO EMCV as a tool for later studies, we investigated the effect of the 2B*KO mutations on viral replication using a GFP-tagged virus (GFP WT EMCV) (14), which contains the GFP ORF immediately followed by the EMCV 3C protease cleavage site, upstream of the leader and capsid proteins. The essential GDD motif in the viral RNA polymerase, 3D, was mutated to GNN to create a replication-incompetent control. This control allowed us to account for potential carryover of the T7 *in vitro*-transcribed RNA, transfected to create the virus stocks, which could technically contribute to GFP levels during initial translation. The GNN mutation and the 2B*KO mutation were independently introduced into the parental genome, creating GFP GNN EMCV and GFP 2B*KO EMCV, respectively. Following transfection, harvesting, and infection, the increasing levels of GFP in an infected cell would be directly proportional to the amount of viral protein produced and hence a reflection of viral replication.

BHK-21 cells were infected with GFP WT EMCV and GFP 2B*KO EMCV, and viral replication over time was measured by live-cell imaging (Fig. 1C). The supernatant from GFP GNN EMCV transfected cells was included as an additional control (GFP GNN EMCV; Fig. 1C). Green calibrated units (GCUs) at each time point were normalized to the GCU in that sample at 5.5 hpi, which was when GFP was first observed in most samples (presumably, this was when viral translation had produced detectable levels of GFP; prior to this, infections were indistinguishable from mock-infected cells). As cell lysis occurred at approximately 8 hpi (one viral replication cycle), GFP was not measured past this point. The fold change in GCU was not significantly different between cells infected with GFP WT EMCV or GFP 2B*KO EMCV at any of the time points measured (Fig. 1C), while GFP GNN EMCV consistently gave only background levels of GCU.

To confirm these results, we introduced the 2B*KO mutation into a WT EMCV replicon (kindly provided by Prof. Ann Palmenberg) (15), where the capsid proteins have been replaced with a firefly luciferase reporter gene. Luciferase activity from BHK-21 cells transfected with *in vitro*-transcribed replicon RNA did not differ significantly between WT and 2B*KO EMCV replicons (Fig. S1). Thus, we were confident that the 2B*KO mutation did not affect viral replication. Therefore, any differences found between 2B*KO EMCV and WT EMCV in subsequent experiments are unlikely to be caused by defects in either PRF or viral replication and are likely to be solely attributable to the loss of 2B* protein.

### Characterization of a virus expressing HA-tagged 2B*

**Fig 2:**
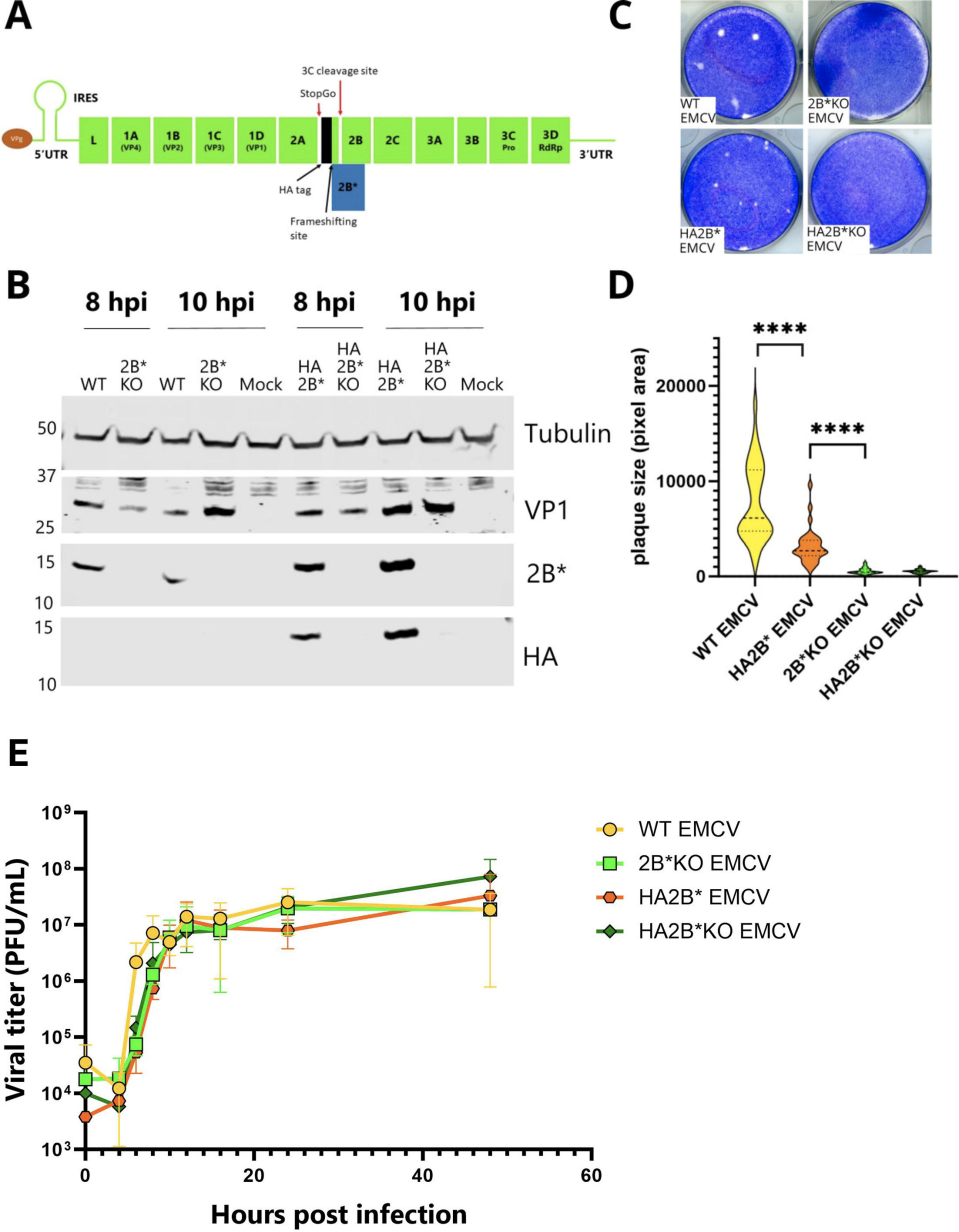
Characterization of HA2B* EMCV. (**A**) Schematic representation of the EMCV viral genome with sequence encoding the HA tag inserted to tag 2B*. (**B**) BHK-21 cells were infected with WT EMCV, 2B*KO EMCV, HA2B* EMCV, or HA2B*KO EMCV at an MOI of 5.0. Cells were harvested at the indicated time points and frozen in complete RIPA buffer. Lysates were subjected to SDS-PAGE and immunoblotting. The membrane was cut at the 25 kDa marker on the ladder to allow the same membrane to be probed by all four antibodies. The molecular mass scale (kDa) is indicated at the left, and antibodies used are labeled at the right. (**C**) Images of plaques formed by the indicated viruses, grown in BSR cells. Images are representative of three independent biological repeats. (**D**) Size distribution of plaques formed by WT EMCV, HA2B* EMCV, and their respective 2B*KO mutants. Distributions shown are based on area measurements of 30 plaques, sampled from three biological repeats. Horizontal lines represent the median (dashed) and upper and lower quartiles (dotted). Statistical analysis (ratio-paired *t*-test): ****P ≤ 0.0001. (**E**) BHK-21 cells were infected with the indicated viruses at an MOI of 3. At the indicated time points post-infection, released and intracellular virus was harvested, and the titer of each was determined by plaque assay. Total infectious virus (PFU/mL) was calculated for each virus at each time point. Data represent the means ± SEM of two independent biological repeats.

Next, we modified the EMCV genome to encode 2B* with an N-terminal HA tag (HA2B* EMCV). This would allow 2B* to be immunoprecipitated from infected samples, and the subsequent identification of its binding partners. The HA tag and a short Gly-Ser flexible linker were encoded immediately following the final proline of the StopGo sequence, responsible for separating 2A from 2B/2B* (Fig. 2A). A similar approach has been previously described to tag 2B* with a V5 epitope (12). As 2B and 2B* share the first 12 residues prior to the change in reading frame, both 2B and 2B* will be translated with the N-terminal HA tag. However, the viral 3C protease is thought to cleave the N terminus of 2B, thereby removing the tag (1, 5, 12). Cleavage has been confirmed by mass spectrometric detection of the cleaved N-terminal fragment using a tagged TMEV cardiovirus, and analyses of radiolabeled translation products (for TMEV) and immunodetected products (for EMCV) have indicated that most or all of 2B is cleaved (5, 12). As Finch et al. (5) reported the identification of peptides consistent with N-terminally cleaved 2B, we were confident that the majority of HA tags would be removed from the 2B protein in our experiments, despite the overlapping nature of the 2B and 2B* ORFs. The KO mutations were also introduced into HA2B* EMCV to create an equivalent KO mutant virus, as an additional control for use in subsequent anti-HA immunoprecipitations. At both 8 and 10 hpi, HA2B* EMCV produced detectable levels of HA2B* in BHK-21 cells (Fig. 2B). Notably, HA-tagged 2B was not detected in any of the lysates (predicted molecular mass 13 kDa), consistent with the HA epitope being cleaved from the majority of 2B.

To ensure that the addition of the HA tag did not functionally disable 2B* and that HA2B* KO EMCV recreated the small plaque phenotype associated with the loss of 2B* (2, 3), the plaques produced by each virus at 48 hpi in BSR cells were measured (Fig. 2C and D). Although HA2B* EMCV created smaller plaques than untagged WT EMCV (median areas 2,694 and 6,140 pixels, respectively) (Fig. 2D), they were still significantly larger than those of 2B*KO EMCV (median area 451 pixels). The decreased size of HA2B* EMCV plaques indicated that the addition of the tag did somewhat affect 2B* function. However, this change was modest, and as the plaques were still substantially larger than those created by 2B*KO EMCV, it is likely that HA-tagged 2B* still interacts with the relevant factors required for the large plaque phenotype. As plaque size results from the combined effects of RNA replication, egress, and entry across adjacent cells, to complement these results, we also assessed the replication kinetics of HA2B* EMCV and HA2B*KO EMCV in BHK-21 cells via a single-step growth curve. This confirmed that they performed similarly to their untagged parental viruses (Fig. 2E). Therefore, HA2B* EMCV was deemed a suitable tool to characterize interaction partners of 2B*.

### Mass spectrometry identifies 14-3-3 protein family members as putative interaction partners of 2B*

**Fig 3:**
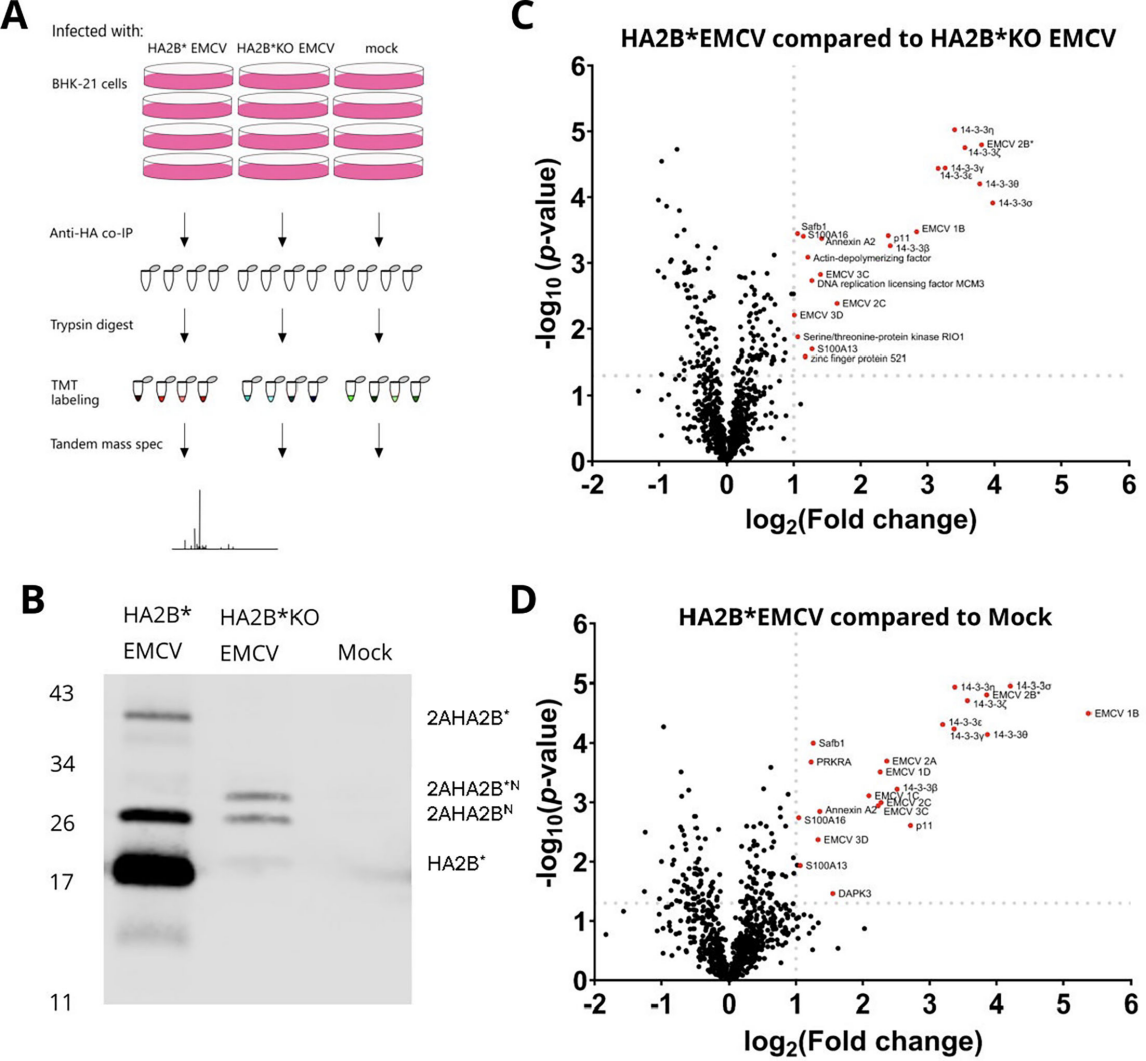
Putative interaction partners of 2B* include the entire family of 14-3-3 proteins. (**A**) Schematic diagram of the TMT experiment. BHK-21 cells were infected with either HA2B* EMCV or HA2B*KO EMCV, or mock infected. At 7 hpi, HA2B* and any interaction partners were purified from the lysates by anti-HA co-immunoprecipitation; 87.5% of each sample was then used for trypsin digestion, TMT labeling, and analysis by mass spectrometry. (**B**) The remaining 12.5% of each co-immunoprecipitated sample was probed for HA-tagged proteins by western blot using an anti-HA antibody. The molecular mass scale (kDa) is indicated at left, and HA-tagged proteins are labeled at right. (**C** and **D**) Two-sample *t*-tests were used to compare protein enrichment in the HA2B* EMCV-infected samples relative to HA2B*KO EMCV-infected samples (panel C) and mock-infected samples (panel D). Candidate interaction partners were defined as those with −log_10_(*P*-value) greater than 1.3 (i.e., *P*-value > 0.05) and log_2_(fold change) > 1 (i.e., fold change greater than 2) and are indicated with red dots and labeled. Note that, due to the presence of background noise, the infected versus mock fold changes for virus proteins are not infinite.

To identify the host and viral protein interactions of 2B*, HA2B* was immunoprecipitated from lysates of HA2B* EMCV-infected BHK-21 cells harvested at 7 hpi in quadruplicate. Immunoprecipitated samples were then subjected to trypsin digestion and labeling by tandem-mass-tagging (TMT) prior to being analyzed by liquid chromatography with tandem mass spectrometry (LC-MS/MS) (Fig. 3A). While the predominant tagged viral protein was 2B*, the StopGo mechanism is approximately 94% efficient in EMCV, and therefore, small amounts of HA-tagged 2A-fusion products (2AHA2B* and 2AHA2B^N^, where 2B^N^ represents the N-terminal fragment of 2B upstream of the 3C protease cleavage site [12]) were produced (Fig. 3B). To ensure any interacting proteins bound to these fusion products could be identified and removed from analysis, HA2B*KO EMCV-infected controls were included in addition to mock-infected controls.

Seventeen proteins were enriched in HA2B* EMCV-infected samples relative to both HA2B*KO EMCV-infected and mock-infected samples, including HA2B* itself (Table 1; Fig. 3C and D). Candidate interaction partners were defined as those having a fold change > 2 and both a Student’s *t*-test difference greater than 1.0 and a −log_10_(*P*-value) greater than 1.3 (i.e., *P* < 0.05). Interestingly, this included the entire family of 14-3-3 proteins. The 14-3-3 family is a group of scaffold proteins involved in a wide range of cellular functions, including apoptosis (16, 17), cell cycle progression (18, 19), nuclear trafficking (20), innate immune signaling (21, 22), autophagy (23), and proteasome function (24). Due to the importance of these proteins in many pathways which EMCV utilizes, we chose to focus on these proteins for subsequent analyses.

**TABLE 1:** Proteins that were statistically significantly enriched in HA2B* EMCV-infected samples relative to both HA2B*KO EMCV-infected and mock-infected samples

Protein	Log_2_(fold change WT/KO)	−Log_10_(Student’s *t-*test *P*-value) WT/KO	Log_2_(fold change WT/mock)	−Log_10_(Student’s *t-*test *P*-value) WT/mock
EMCV 3D	1.01	2.21	1.33	2.37
Safb1	1.06	3.45	1.26	4.00
S100A16	1.14	3.41	1.04	2.74
S100A13	1.27	1.70	1.06	1.93
EMCV 3C	1.40	2.83	2.23	2.94
Annexin A2	1.42	3.37	1.36	2.85
EMCV 2C	1.65	2.39	1.65	2.39
Calpactin I light chain (S100A10)	2.41	3.41	2.71	2.61
14-3-3β	2.44	3.26	2.51	3.22
EMCV 1B	2.84	3.47	5.37	4.50
14-3-3ε	3.16	4.44	3.19	4.31
14-3-3γ	3.26	4.44	3.37	4.24
14-3-3η	3.40	5.02	3.38	4.94
14-3-3ζ	3.56	4.75	3.56	4.71
14-3-3θ	3.78	4.20	3.86	4.14
EMCV 2B*	3.81	4.80	3.85	4.81
14-3-3σ	3.98	3.91	4.21	4.96

**Fig 4:**
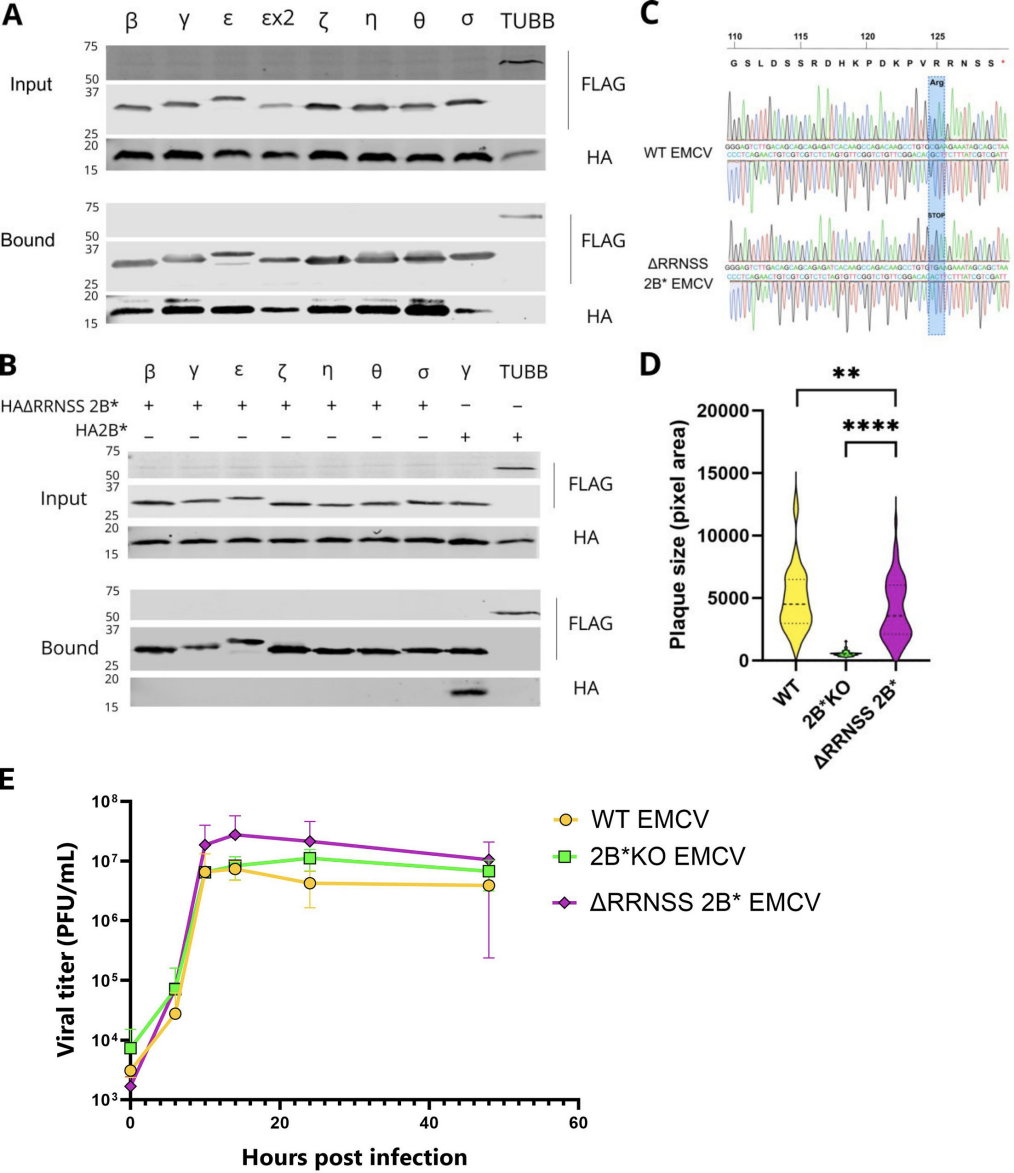
2B* binds to the entire family of 14-3-3 proteins via a C-terminal RRNSS sequence. (**A**) BHK-21 cells were co-transfected with equal amounts of pCAGG-HA2B* and the specified N-terminally FLAG-tagged 14-3-3 encoding plasmid (pCAGG-FLAG 14-3-3x), 24 h prior to immunoprecipitation via the FLAG epitope. Samples were then subjected to SDS-PAGE and immunoblotting using the indicated antibodies. Data shown are representative of two independent biological repeats. (**B**) BHK-21 cells were co-transfected with both a plasmid encoding either an N-terminally FLAG-tagged 14-3-3 protein or tubulin β chain and another encoding either HAΔRRNSS2B* or HA2B*, as indicated. Cell lysates were immunoprecipitated with anti-FLAG antibody and subjected to SDS-PAGE and immunoblotting. Data shown are representative of two independent biological repeats. (**C**) Confluent monolayers of BHK-21 cells were infected with WT EMCV or ΔRRNSS 2B* EMCV at an MOI of 0.01. At 24 hpi, RNA was extracted from each sample and subjected to RT-PCR to amplify the region of interest. The cDNA was sequenced (Sanger method) with both forward and reverse primers. Both chromatograms have only one clear peak for each nucleotide, indicating each infected sample contained only one EMCV sequence detectable by Sanger sequencing. Chromatograms shown are representative of three independent biological repeats. (**D**) BSR cells were infected with the indicated viruses 1 h prior to being overlaid with semi-solid medium (1% LMA) for 48 h. Distributions shown are based on area measurements of 60 randomly chosen plaques sampled over three biological repeats. Horizontal lines represent the median (dashed) and upper and lower quartiles (dotted). Statistical analysis (ratio-paired *t*-test): **P ≤ 0.01 and ****P ≤ 0.0001. (**E**) MEF cells were infected with the indicated viruses at an MOI of 5. At the indicated time points post-infection, released and intracellular virus was harvested, and the titer of each was determined by plaque assay. Total infectious virus (PFU/mL) was calculated for each virus at each time point. Data represent the means ± SEM of two independent biological repeats.

To confirm their interaction with 2B*, a construct expressing HA2B* was co-transfected into BHK-21 cells along with each construct encoding an N-terminally FLAG-tagged 14-3-3 protein, cloned from BHK-21 cDNA. As 14-3-3ε isoforms X1 and X2 could not be distinguished by the peptides detected by mass spectrometry, both were included. The 14-3-3 proteins were then immunoprecipitated from the samples via the FLAG tag. Immunoprecipitated samples were subjected to SDS-PAGE and immunoblotting to determine whether HA2B* was bound to these putative interaction partners (Fig. 4A). As the tubulin β-chain was not identified by the co-immunoprecipitation mass spectrometry analysis as a potential interaction partner of HA2B*, a construct encoding N-terminally FLAG-tagged tubulin β-chain (FLAG-TUBB) was included as a negative control. The interaction between 2B* and all of the 14-3-3 proteins was verified as the FLAG-tagged proteins were all found to co-precipitate HA2B* (Fig. 4A). These proteins are therefore able to bind to 2B* both during infection and in overexpression, and thus, other viral proteins are not required to mediate this interaction. Although the other putative interaction partners (Table 1) were also investigated by reciprocal (anti-FLAG) immunoprecipitation, only annexin A2 and calpactin I light chain could be confirmed to interact with 2B* via this method (Fig. S2). These interactions were not investigated further in this study.

### 14-3-3 binding is mediated by a C-terminal motif in 2B* and is not responsible for the 2B*KO small plaque phenotype

To identify the motif(s) within 2B* responsible for 14-3-3 binding, we utilized the online resource Eukaryotic Linear Motifs (ELM) (25), which searches for short linear motifs corresponding to known protein interaction sites. The ELM tool predicted the C-terminal RRNSS sequence of 2B* to be a binding site for 14-3-3 proteins. Indeed, this was the best-scoring potential interaction site in 2B*, with a reported *P*-value of 6.4 × 10^−5^. The C-terminal RRNSS sequence is highly conserved across EMCV strains with occasional variations in the last position (to N, I, or L) (7). The 14-3-3 binding motifs fall into three groups, allowing mode I, mode II, and mode III binding with their respective ligands. All three binding motifs are highly conserved (26). Mode III binding sites are invariably located at the C terminus of the binding partner in question, with the essential motif being p[S/T]-X_1–2_-COOH with upstream arginine residues preferred (RRXp[S/T]-X_1–2_-COOH) (where p represents one phosphate group) (27, 28). The 14-3-3 proteins form homo- and heterodimers, binding the target protein partner when the penultimate serine (within the mode III recognition motif) is phosphorylated (27–29), leading to translocation or sequestration of the binding partner.

To investigate whether the C-terminal RRNSS sequence is indeed required for 2B*:14-3-3 binding, a truncated HA-tagged 2B* construct (HAΔRRNSS 2B*) was engineered using a R125Stop mutation, thus removing the five C-terminal residues of 2B*. This construct was co-transfected into BHK-21 cells alongside each construct encoding a FLAG-tagged 14-3-3 protein, and the FLAG co-immunoprecipitation assay was repeated (Fig. 4B). Every FLAG-tagged member of the 14-3-3 family was entirely unable to bind to the co-transfected ΔRRNSS2B* mutant, whereas the WT HA2B* was still successfully bound by a representative 14-3-3 protein, 14-3-3γ. This clearly demonstrates that the RRNSS sequence of 2B* is essential for the 2B*:14-3-3 interaction, and deletion of this sequence completely prevents this interaction.

Confocal microscopy confirmed that WT 2B* and all 14-3-3 family members were dispersed throughout the cytosol (Fig. S3 to S5), and redistribution was not observed during co-expression. Removing the 2B* C-terminal motif did not alter this cytosolic distribution of either 2B* or two representative 14-3-3 proteins during co-expression (Fig. S6).

We next investigated whether the lack of the 14-3-3:2B* interaction contributes to the small plaque phenotype characteristic of 2B* KO viruses (2, 3). The R125Stop mutation, which is synonymous in the 2B reading frame, was introduced into the viral genome, creating ΔRRNSS 2B* EMCV. To confirm that this mutation did not revert during infection, the virus produced from infected BHK-21 cells was sequenced by Sanger sequencing (Fig. 4C). No detectable reversion was observed by 24 hpi. Interestingly, the small plaque phenotype associated with 2B*KO EMCV (median plaque area 524 pixels) was not reproduced by ΔRRNSS 2B* EMCV (Fig. 4D). Although the difference between WT EMCV and ΔRRNSS 2B* EMCV plaque sizes was still statistically significant, it was very modest (median areas 4,516 and 3,577 pixels, respectively). Therefore, the 2B*:14-3-3 interaction is unlikely to contribute to the increased plaque size seen in WT EMCV infection, indicating that this interaction does not drive the lytic cell death pathways that 2B* promotes (7). A single-step growth curve also indicated that ΔRRNSS 2B* EMCV does not exhibit significantly altered replication kinetics compared to WT EMCV (Fig. 4E).

### The 2B*:14-3-3 complex follows a two-site binding model

**Fig 5:**
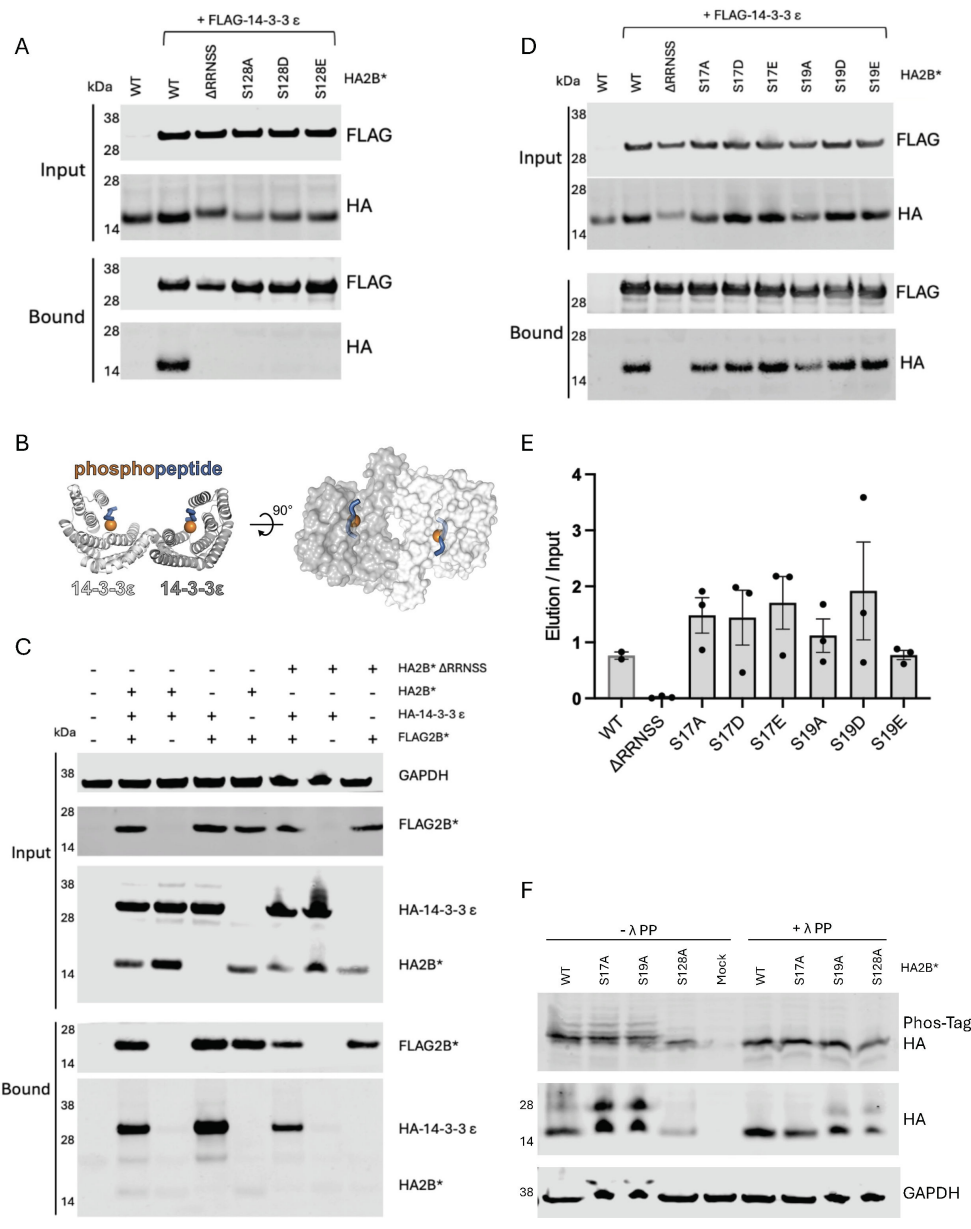
2B* forms a ternary complex with a 14-3-3 dimer and the phosphorylated residue S128. (**A**) BSR cells were co-transfected with both pCAGG-FLAG 14-3-3ε and one of pCAGG-HA2B*, pCAGG-HAΔRRNSS2B*, or the indicated mutants of S128 (S128A/D/E) in the pCAGG-HA2B* backbone. Cell lysates were immunoprecipitated with anti-FLAG antibody and subjected to SDS-PAGE and immunoblotting. Data shown are representative of three independent biological repeats. (**B**) Dimer of human 14-3-3ϵ (PDB 2BR9; gray) (30) in complex with a consensus phosphoserine peptide (Pep I; blue ribbons). Phosphate groups of phosphoserine residues are shown as spheres. 14-3-3ϵ protomers are shown as ribbons (left) and semi-transparent molecular surfaces in an orthogonal view (right). (**C**) BSR cells were co-transfected with various combinations of pCAGG-HA2B*, pCAGG-HAΔRRNSS2B*, pCAGG-FLAG2B*, pCAGG-HA 14-3-3ε, and empty vector. Cell lysates were immunoprecipitated with anti-FLAG antibody and subjected to SDS-PAGE and immunoblotting. Data shown are representative of three independent biological repeats. (**D**) BSR cells were co-transfected with both pCAGG-FLAG 14-3-3ε and one of pCAGG-HA2B*, pCAGG-HAΔRRNSS2B*, or the indicated mutants of S17 (S17A/D/E) or S19 (S19A/D/E) in the pCAGG-HA2B* backbone. Cell lysates were immunoprecipitated with anti-FLAG antibody and subjected to SDS-PAGE and immunoblotting. Data shown are representative of two (WT) or three (all mutants) independent biological repeats. (**E**) Quantification of HA2B* immunoprecipitation efficiency from panel **D**, displayed as the ratio of elution to input HA-tagged protein band intensity. Data represent the mean ± SEM of two (WT) or three (mutants) biological repeats. (**F**) BSR cells were transfected with pCAGG-HA2B* or mutants thereof containing S17A, S19A, or S128A. Lysates were harvested and either treated with λ protein phosphatase (PP) or mock treated, prior to electrophoretic separation on a 15% acrylamide gel supplemented with 50 µM Phos-Tag acrylamide (upper panel). Cell lysates were also subjected to standard SDS-PAGE and immunoblotting (center and lower panels). One of three experimental repeats is shown. Additional HA-specific higher-order bands (presumably undenatured complexes) are not observed in repeat experiments (see Fig. S9 for additional images).

To further validate the mode III binding motif of 2B*, we engineered point mutations into pCAGG-HA2B*, replacing the presumably phosphorylated serine at position 128 with phosphoablative (S128A) or phosphomimetic residues (S128D and S128E). None of the mutant 2B* proteins were able to bind to a representative 14-3-3 protein, as evidenced by a lack of co-immunoprecipitation (Fig. 5A). This confirmed the specificity of the interaction and indicated that a serine is essential at this position.

Given that 14-3-3 homo- or heterodimers have two peptide-binding amphipathic grooves (Fig. 5B), we investigated whether two molecules of 2B* associate with a homodimer of 14-3-3ε to form a quaternary complex by co-transfecting various combinations of constructs encoding HA2B*, FLAG2B*, and HA-14-3-3ε and immunoprecipitating via the FLAG tag. Surprisingly, HA2B* was not co-immunoprecipitated with FLAG2B*, regardless of the presence of HA-14-3-3ε (Fig. 5C). The reciprocal immunoprecipitation produced similar results, with no FLAG2B* visibly co-immunoprecipitating with HA2B* via the HA tag (Fig. S7). This is consistent with 2B* binding 14-3-3 with a 1:2 stoichiometry, whereby single HA2B* or FLAG2B* molecules (but not both) engage with a dimer of 14-3-3. This would indicate the existence of two spatially distinct 14-3-3 binding sites within 2B*, one being the C-terminal five residues (RRNSS). Such “two-site” 14-3-3 binding proteins are being increasingly reported in the literature (26, 27, 31–35).

Structure predictions of a 14-3-3ϵ homodimer in complex with a single molecule of 2B*, where the penultimate serine residue (S128) was modeled as phosphoserine, were performed using AlphaFold3 (36) and the 23 known unique 2B* sequences (7). While much of 2B* was predicted with very low confidence, consistent with the protein being predominantly unstructured, all 23 predicted models placed the C-terminal region of 2B* in the amphipathic groove of one 14-3-3ϵ protomer with moderate confidence (Fig. S8A). In 18/23 models, a short helical region (residues 100–111) was predicted with moderate confidence to interact with helices α6 to α8 on the opposite face of the same 14-3-3ϵ protomer (Fig. S8B). Interestingly, in 13/23 models, residues 16–19 of 2B* were predicted to occupy the amphipathic groove of the opposing 14-3-3ϵ protomer, albeit with relatively low confidence (Fig. S8C). Since this sequence region contains two highly conserved serine residues (7), we used AlphaFold3 to predict the structure of a 14-3-3ϵ homodimer in complex with 2B*, where serine residues 128 and either 17 or 19 were modeled as phosphoserines (Fig. S8D). While much of the 2B* protein is still predicted with low confidence, the N-terminal and C-terminal phosphopeptide motifs of 2B* are predicted to bind amphipathic grooves of the 14-3-3ϵ heterodimer with moderate confidence. Visual inspection of the 2B* model, where residues 19 and 128 were modeled as phosphoserine, indicated that this N-terminal region is similar to the mode I binding motif (canonical motif: RSXpSXP, EMCV 2B*: _16_RSGSVI_21_) (29, 37, 38).

Structurally informed mutagenesis was then used to test the contributions of serine residues 17 and 19 to 14-3-3 protein binding. After mutating S17 or S19 to alanine, glutamic acid, or aspartic acid in the pCAGG-HA2B* vector, these HA2B* constructs were co-transfected alongside FLAG-14-3-3ϵ into BSR cells, before immunoprecipitation via the FLAG tag at 24 h post-transfection. While mutation of either residue to alanine appeared to yield a modest reduction in binding (Fig. 5D), quantitative analysis of the ratio of immunoprecipitated versus input HA2B* showed that these changes were not statistically significant (Fig. 5E). This suggests that either the contribution of these residues to binding is modest and not measurable with immunoprecipitation or that they do not contribute.

It is generally assumed that all modes of 14-3-3 binding rely upon phosphorylation of the interacting peptide, at a serine or threonine located within the binding groove of each 14-3-3 monomer (although non-phosphorylated ligands are known, see Discussion). We therefore analyzed the phosphorylation status of the mutant 2B* proteins (Fig. 5F; Fig. S9) to investigate whether serine residues at positions 17, 19, and 128 were phosphorylated in the WT protein. BSR cells were transfected with various HA2B* constructs, and lysates were phosphatase treated at 24 h post-transfection. Samples were electrophoresed on Phos-Tag gels to separate differentially phosphorylated protein species, prior to standard immunoblotting for the HA tag. SDS-PAGE was conducted alongside to ensure equivalent protein loading. These results indicated that while S128 appeared to be phosphorylated, no other phospho-isoforms of 2B* were detectable in this system. Taken together, our data are consistent with 2B* containing a C-terminal mode III motif where phosphoserine at position 128, but not phosphomimetic residues glutamate or aspartate, supports 14-3-3 binding.

### The 2B*:14-3-3 interaction interferes with innate immune signaling outside the context of infection

Presumably, the 2B*:14-3-3 interaction performs a function that is beneficial to the virus, although not essential as we saw no evidence of increased replication rates when the interaction motif was intact compared to the C-terminally truncated 2B* (Fig. 4E). We hypothesized that the sequestration of 14-3-3 proteins by 2B* may contribute to the evasion of innate immune signaling as other viral binding partners of 14-3-3 are known to have similar effects (8–11). During viral infection, RIG-I is relocalized from the cytosol to the mitochondria by 14-3-3ε, which forms a complex with both RIG-I and TRIM25 (22), the ubiquitin ligase essential for the antiviral function of RIG-I (39), enabling the activation of MAVS on the mitochondrial membrane. In addition, 14-3-3η is responsible for the redistribution of MDA5, enabling MDA5-induced antiviral IFN-β signaling via MAVS (21). It is unclear whether 14-3-3 proteins bound to 2B* would be able to perform these functions. Therefore, we next investigated the ability of 2B* to antagonize innate immune signaling via the 14-3-3 interaction.

**Fig 6:**
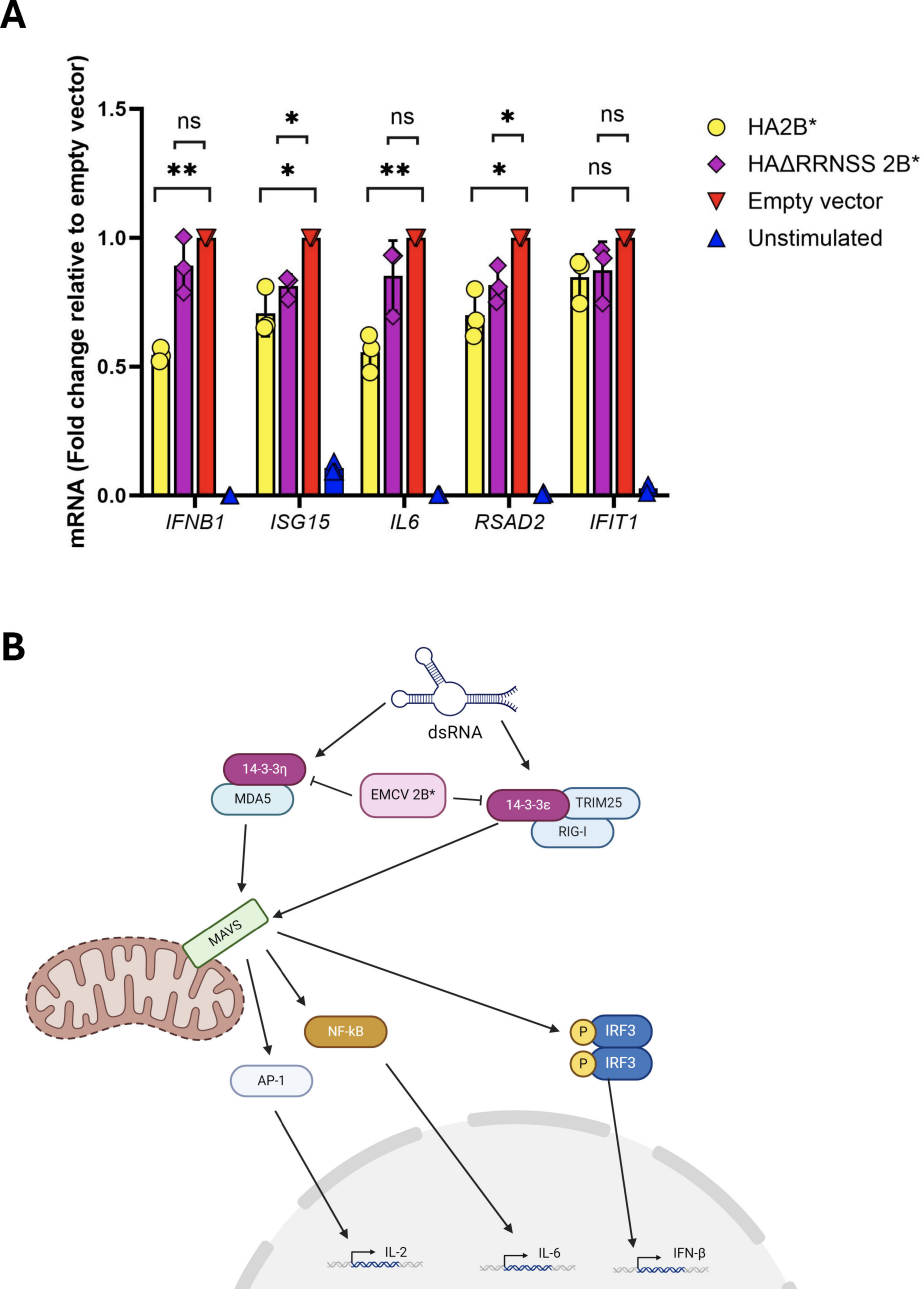
Overexpressed 2B* interferes with innate immune signaling via its interaction with 14-3-3 proteins. (**A**) MEF cells were transiently transfected with constructs encoding HA2B* or HAΔRRNSS 2B*, or the empty vector, as indicated 24 h prior to transfection with poly(I:C) (final concentration 10 ng/mL) for 6 h. Unstimulated, empty vector-transfected controls were also included. The relative expression level of each gene was determined by qRT-PCR. Expression levels were normalized internally to GAPDH and poly(I:C)-stimulated samples transfected with the empty vector (pCAGG). Data shown are the mean ± SD of three biological repeats. Statistical analysis (unpaired Welch *t*-test): ns, not significant; *P ≤ 0.05; and **P ≤ 0.01. (**B**) 2B* may reduce NFκB and IRF3 signaling by inhibiting the translocation of innate immune signaling molecules MDA5, RIG-I, and TRIM25 by preventing their interactions with 14-3-3 proteins, thus reducing MAVS activation.

MEF cells were transiently transfected with pCAGG-HA2B*, pCAGG-HAΔRRNSS2B*, or empty pCAGG 24 h prior to the activation of the IRF3 and NFκB signaling pathways by transfection with poly(I:C), a dsRNA mimic (P1530, Sigma). These cells are known to mount a functional, intact interferon response during viral infection (40–42). After 6 h, *IFNB1*, *ISG15*, *IL6*, *RSAD2,* and *IFIT1* transcript levels were quantified by qRT-PCR, normalized to GAPDH mRNA using the ΔΔCT method, and compared to those for the empty pCAGG-transfected samples. While overexpression of HA2B* prior to poly(I:C) stimulation significantly reduced the transcription of *IFNB1*, *IL6,* and the ISGs *ISG15* and *RSAD2* involved in innate immune signaling (Fig. 6A), this antagonistic ability was abrogated by the loss of the RRNSS motif responsible for 14-3-3 binding, indicating that the 14-3-3 binding capability of 2B* is essential for this function. However, it must be highlighted that this experiment assessed the effects of 2B* in isolation, outside of an EMCV infection where it may be influenced by other viral proteins and would be in a cellular landscape modified by viral infection. Equally, a detailed molecular mechanism of how 2B* sequestration of 14-3-3 proteins blocks innate immune signaling has yet to be established, although it can be hypothesized that the interaction with 2B* would prevent the 14-3-3 proteins from translocating RIG-I and MDA5 to MAVS to initiate the innate immune response (Fig. 6B).

## DISCUSSION

In this study, we engineered an EMCV genome that encodes an HA-tagged 2B* protein, enabling the first characterization of the binding partners of 2B*. While the addition of the tag had a modest effect upon plaque size, it did not completely abrogate 2B* function (Fig. 2C and D). We also confirmed that the 2B*KO virus had WT levels of PRF and RNA replication, providing future studies with a tool for studying 2B* phenotypes without the problem of confounding effects (Fig. 1B and C; Fig. S1). We identified 17 putative binding partners of 2B* (Fig. 3; Table 1), 7 of which are members of the 14-3-3 family, and determined the C-terminal five residues of 2B* as being essential for the 2B*:14-3-3 interaction (Fig. 4), forming a mode III interaction motif that contains a phosphorylated serine residue (Fig. 5).

The 14-3-3 proteins are a family of scaffold proteins that form both homo- and heterodimers, performing a plethora of roles. Hence, many pathways may be affected by 14-3-3 interactions with 2B*. As scaffold proteins possessing two ligand-binding pockets, it has been extensively suggested in the literature that a 14-3-3 dimer may bind simultaneously to two different protein partners, either bringing them together or sequestering them to regulate protein:protein interactions (16, 18, 20, 26, 43, 44). While this is certainly possible, there is a dearth of specific examples of such quaternary complexes. It is clear, however, that many homodimeric protein ligands bind to 14-3-3 dimers in a 2:2 stoichiometry, in a phosphorylation-dependent manner, leading to indirect regulation of their availability and enzymatic activity by various kinases and phosphatases (45–48).

In addition to this conventional model, there is a growing body of literature describing “two-site” binding partners; that is, proteins that possess two intra-chain 14-3-3 binding motifs (26, 27, 32–35). These may be either tandem sites, located approximately 20 residues apart (33, 35, 49), or distal sites, separated by an intrinsically disordered domain of hundreds of residues (27, 32). Indeed, 14-3-3 proteins are generally regarded to bind to disordered regions and induce localized regions of structure in their partners (34, 44). This may be the case for 2B*, as its C terminus is predicted to be intrinsically disordered when unbound (by analysis with AlphaFold, I-TASSER, and NetSurfp).

There is no clear correlation between the occurrence of a particular binding mode (I, II, or III) and whether a target protein possesses one or two binding sites; however, there have been suggestions that many known 14-3-3 binding proteins may actually possess a single, optimal site (the so-called gatekeeper site) in addition to a secondary, lower-affinity site (33, 50). Such gatekeeper sites may be either mode I or II motifs (33) or C-terminal mode III motifs (27). Often, phosphomimetic residues are unable to successfully replace phosphorylated serines in these optimal binding motifs (43), consistent with our S128D/E results. Non-canonical motifs are also common within dual binding proteins (26), and notably, the secondary motifs are often not essential for binding of the target to the 14-3-3 dimer but significantly increase binding affinity (33). In the case of 2B*, it is clear that binding relies upon a phosphorylated S128 within a mode III motif. However, a secondary, non-canonical binding site presumably exists, given the inability of differently tagged 2B* proteins to co-immunoprecipitate during overexpression.

Despite the high conservation of serine residues 17 and 19 (7), they were not essential for 14-3-3 binding. It is intriguing that, upon visual inspection, mutation of these residues often resulted in a lower abundance of 2B* in transfected cells as evidenced by less HA-tagged protein in the input samples (Fig. 5A and D). While it remains possible that this merely reflects experimental variability, this was reproducible and may indicate decreased stability of 2B* in the absence of the 14-3-3 scaffold. 14-3-3 dimers are known to stabilize their interaction partners (16, 51), and similar observations have been reported for mutants of other viral 14-3-3 binding proteins (52).

Our evidence suggests that overexpressed 2B* has only one phospho-isoform (being phosphorylated at S128), indicating that, if S17 and S19 do support 14-3-3 binding, either it is via a non-canonical, non-phosphorylated peptide or that 2B* is differentially phosphorylated in the context of infection. Although uncommon, non-phosphorylated 14-3-3 binding ligands are known, although they tend to contain phosphomimetic residues (9, 53–57). There are also reports of phosphorylation-independent binding, whereby phosphorylation is not essential for peptide:14-3-3 binding but increases affinity (58–60). Finally, there is also a single report of 14-3-3 recognition of an acetylglucosamine-linked serine, in place of a phosphorylation modification, whereby the glycopeptide occupies the ligand binding groove (61). Other post-translational modifications such as this were not investigated in our study.

Given the vast range of cellular processes to which 14-3-3 proteins contribute, the effects of the 2B*:14-3-3 interaction may be numerous and far-reaching. One potential functional consequence may be the disruption of innate immune signaling, as sequestration of 14-3-3 proteins would presumably prevent their contribution to MDA5 and RIG-I activation (21, 22) and subsequent inflammatory cytokine induction. Here, we present preliminary data to support this hypothesis (Fig. 6A), although a detailed molecular mechanism has yet to be established.

Although the involvement of 14-3-3 proteins in antiviral immunity, through interactions with RIG-I (22, 62), MDA5 (21), and TRIM25 (10), is a fairly recent discovery in the long list of 14-3-3 functions, already several viruses have been found to target these interactions, enhancing viral replication through 14-3-3 sequestration or degradation. Investigating whether the mechanism of action of 2B* mimics any of these will be a subject for future work. While it is tempting to speculate on functional conservation across the *Picornaviridae*, as the proteases of multiple enteroviruses were found to cleave 14-3-3ε and reduce its interaction with RIG-I (11), we saw no evidence of this during EMCV infection. Rather than cleavage, 2B* may instead prevent 14-3-3-mediated translocation of RIG-I to the mitochondrial membrane (similarly to the influenza A virus protein NS1 [8])

The fact that 2B* is able to bind to all 14-3-3 family members (Fig. 4A) indicates a highly specific mechanism of action is improbable, and broader effects, perhaps targeting multiple RLRs, are more likely. The most logical parallel is perhaps therefore that of the Zika virus NS3 protein, which was found to bind to both 14-3-3ε and 14-3-3η via a single non-canonical, non-phosphorylated RLDP motif, efficiently preventing both RIG-I and MDA5-mediated signaling (9). Related to this, it must be noted that the poly(I:C) utilized in our work may induce both RIG-I and MDA5 signaling due to the variable length (200–1,250 bp, according to the supplier). As such, we cannot infer which pathway is being preferentially activated at this stage, although EMCV is generally thought to primarily induce MDA5-dependent signaling (21). Interestingly, EMCV has been used as a model virus to study the interactions of 14-3-3η with MDA5 (21).

As the presence of 2B* appeared to reduce the levels of all antiviral transcripts tested (Fig. 6A), we cannot eliminate the possibility that the 2B*:14-3-3 interaction causes a global reduction in transcription instead of specifically influencing the antiviral response. However, 2B* does not have a nuclear localization (Fig. S3 to S6) and, although the 14-3-3 proteins are involved in nuclear transport of some transcription factors (20, 63), these do not include IRF3 or NFκB, which are responsible for the upregulation of the target genes tested (*IL6, IFNB1, ISG15, IFIT1,* and *RSAD2*). In addition, EMCV-induced reduction of host transcription and translation (so-called host shutoff) occurs from 4 hpi, before 2B* is efficiently translated (12, 64). Therefore, it is most likely that the effect is mediated prior to the nuclear import of IRF3 and NFκB, as the 2B*:14-3-3 interaction would impede the earlier translocation of both RIG-I and TRIM25 by 14-3-3ε (22) and MDA5 by 14-3-3η (21) (Fig. 6B), in a manner similar to the mechanisms exerted by the 14-3-3- binding proteins of influenza A virus, enteroviruses, Zika virus, and Epstein-Barr virus (8–11).

In summary, we propose a model whereby 2B* is able to bind to all 14-3-3 family members, forming a ternary complex with phosphoserine 128 located in one ligand groove of a 14-3-3 dimer. This appears to be an essential “gatekeeper” interaction, while the secondary site is currently unidentified. While this interaction does not contribute to the control of cell death pathways previously ascribed to 2B* and responsible for the small plaque phenotype observed in 2B*KO viruses, it may have an abundance of other effects, one of which may be inhibition of antiviral signaling. Our findings add 2B* to the growing list of viral proteins that interact with the 14-3-3 proteins (8–11) and identify 2B* as another “transframe” protein with a potential role in antagonizing the innate immune response (65–67).

## MATERIALS AND METHODS

### Mammalian cell culture

BHK-21 (ATCC) (baby hamster kidney fibroblast), BSR (single cell clone of BHK-21 cells), and mouse embryonic fibroblast (MEF) cells were maintained in Dulbecco’s Modified Eagle Medium with high glucose (Sigma), supplemented with 10% (vol/vol) heat-inactivated fetal calf serum (FCS), 25 mM HEPES, 2 mM L-glutamine, and non-essential amino acids (Sigma) (“complete DMEM”) in a humidified 5% CO_2_ atmosphere at 37°C. All cell lines were confirmed to be mycoplasma-free at regular intervals (MycoAlert PLUS Assay, Lonza).

### RT-PCR

Uninfected BHK-21 cells or cells infected at an MOI of 0.1 were trypsinized either 24 h after plating or 24 hpi, respectively. The trypsin was neutralized in 10% FCS DMEM before the cells were pelleted at 1,000 × *g,* and the media were removed before the RNA was extracted using a commercial kit as recommended (RNeasy, Qiagen). Reverse transcription of each sample was carried out using 5 µg of RNA, random hexamers, and Superscript III reverse transcriptase (ThermoFisher Scientific). PCR using the relevant primers was performed immediately after reverse transcription.

### Expression plasmids

The pCAGG-HA2B* expression construct was originally synthesized from a gene block (Integrated DNA Technologies), designed with the 2B* coding sequence from the WT EMCV clone, with an additional Kozak sequence, the HA tag, and a GGSGGS linker sequence as well as a non-viral termination codon between *Pac*I and *Bgl*II restriction sites (sequence available upon request). The coding sequence for pCAGG-FLAG2B* was amplified from this template via PCR and cloned into the empty pCAGG vector using *Pac*I and *Bgl*II restriction sites. Serine mutants were engineered by site-directed mutagenesis.

For all cellular proteins identified as being potential interaction partners of 2B* (by affinity capture coupled to quantitative proteomics), corresponding coding sequences were cloned from cDNA of uninfected BHK-21 cells. RNA was extracted from BHK-21 cells, reverse transcribed (see above), and the cDNA was used for PCR amplification. Primers for the PCR amplification of all constructs were designed to add to each gene flanking *Pac*I and either *Afl*II or *Bgl*II restriction sites, as well as an N-terminal FLAG tag separated by a GGSGGS linker (plasmid sequences available upon request). Constructs encoding annexin A2 and calpactin I light chain with a C-terminal FLAG tag were also generated from uninfected BHK-21 cDNA. All amplicons were digested and ligated into pCAGG. The coding sequence for pCAGG HA 14-3-3ε was amplified from the corresponding FLAG-tagged construct.

### DNA transfection

BHK-21 and BSR cells were transfected at 60%–70% confluency in a 6-well plate seeded the day prior. A volume of 15 µL Lipofectamine 2000 (Invitrogen) was added to 300 µL Opti-MEM, and a total of 4 µg DNA was added to another 300 µL Opti-MEM. These solutions were then mixed and incubated for 20 min while the cells were washed with PBS. Following the addition of the transfection mixture, cells were incubated for 3.5 h at 37°C with gentle agitation. A volume of 2 mL of complete DMEM was then added to each well and incubated for 24 h. For all co-immunoprecipitation samples, including those used for TMT mass spectrometry analysis, 10 cm dishes or T75 flasks of BHK-21 or BSR cells were transfected using a scaled protocol, similar to that described above.

### Frameshifting assays

For the WT sequence and each mutant to be tested, 105 nt (106 nt for −1 SS-SL) covering the 2B* frameshift site (11 nt upstream, 7 nt slippery sequence, and 87 nt downstream) was amplified by two-step overlap extension PCR (primer sequences available upon request; numbers exclude restriction enzyme sites). Amplicons were ligated into pSGDLuc via *Bgl*II and *Psp*XI restriction sites. These fragments included the desired mutations for testing, as well as the slippery sequence and stem-loop required for frameshifting (3).

BHK-21 cells were subcultured 24 h prior to being reverse transfected with the pSGDLuc plasmids in a 96-well plate. Upon reverse transfection, cells were trypsinized and washed in DMEM containing 2% FCS, 25 mM HEPES, and 1 mM L-glutamine and washed again in DMEM with 25 mM HEPES and 1 mM L-glutamine without FCS. The cells were then resuspended in this serum-free DMEM. One hundred fifty nanograms of total DNA and 0.5 µL Lipofectamine 2000 per well were incubated at room temperature in 10.5 µL of Opti-MEM (Gibco) for 20 min prior to the addition of 6.5 × 10^4^ BHK-21 cells per well. FCS (5%) was added, and the transfections were immediately seeded into plates in triplicate and incubated for 24 h before being frozen in 50 µL of passive lysis buffer (Promega).

After thawing, 30 µL of the lysate was sequentially mixed with 30 µL of each luciferase reagent (Promega) as described by the manufacturer.

### EMCV replicons

The WT EMCV replicon, Rz-pMluz, was kindly gifted by Prof. Ann Palmenberg (University of Wisconsin-Madison) (15). Note that this replicon has an intact poly(C) tract, whereas the WT EMCV used elsewhere in this work does not. The GNN and 2B*KO mutations were introduced by site-directed mutagenesis. Plasmids were linearized with *BamH*I, and replicon RNA was *in vitro* transcribed using the T7 RiboMax kit (Promega). Transcripts were purified (RNeasy kit, Qiagen) before being reverse transfected into BHK-21 cells in a 96-well plate format. At the indicated times post-transfection, the media were removed, and the cells were frozen in passive lysis buffer. All cells were thawed simultaneously, and firefly luciferase activity was measured (One-Glo Luciferase System, Promega E6110).

### Quantitative RT-PCR

Following transfection of MEF cells, the media were changed prior to the addition of Opti-MEM with Lipofectamine 2000 (Invitrogen) and poly(I:C) to a final concentration of 10 µg/mL poly(I:C). After 6 h, RNA was extracted, and 1 µg of total RNA was used for reverse transcription (Quantitect reverse transcription kit, Qiagen) as per instructions. cDNA was amplified using a ViiA 7 real-time PCR system (ThermoFisher Scientific). The reaction cycle threshold was determined using the following program: (i) initial heating to 55°C for 2 min; (ii) initial denaturation for 10 min at 95°C; (iii) 40 cycles of denaturation for 15 s at 95°C and annealing for 1 min at 60°C. The melt curve was calculated by denaturation for 15 s at 95°C and annealing for 1 min at 60°C with 0.05°C increments to 95°C. The primer sequences for mouse IL6 were (sense) 5′ GAAGTTCCTCTCTGCAAGAGACTTCCATC and (antisense) 5′ GAAGTTCCTCTCTGCAAGAGACTTCCATC. Primer sequences for other transcripts are available upon request and have been previously published (68–70). Primer efficiency was calculated during qRT-PCR optimization, and an efficiency between 90% and 110% was obtained for all primer pairs (data not shown). During data analysis, amplification of each gene was normalized to GAPDH amplification. The fold change of each gene was calculated relative to the empty vector transfected, poly(I:C) stimulated control.

### EMCV reverse genetics

The parental (WT) EMCV sequence has been previously described (3) and is based on the EMCV subtype mengovirus cDNA, pMC0, developed by Ann Palmenberg (University of Wisconsin-Madison) (71). It resembles GenBank accession DQ294633.1, although the poly(C) tract is absent and there are 13 single-nucleotide differences (A2669C, G3044C, C3371T, A4910C, G4991A, C5156T, G5289A, G5314C, G5315A, A5844C, G6266A, G6990A, and A6992G; DQ294633.1 coordinates). The 2B*KO mutation in a subclone has been previously described (12). Regions encompassing the mutations of interest were digested using *Bgl*II and *Pac*I and ligated into the WT EMCV molecular clone.

The GFP-Lzn EMCV clone was a kind gift from Prof. Frank van Kuppeveld and was derived from the clone pRLuc-QG-M16.1 (14, 72). The Lzn mutation was mutated to WT, and the 2B*KO and GNN mutations were subsequently introduced (primer sequences available upon request). All GFP viral clones contain the EMCV IRES followed by the first six codons of the viral protein L, followed by a sequence encoding GFP inserted into restriction enzyme cloning sites. The viral 3C protease cleavage site Gln-Gly immediately precedes the full-length viral polyprotein coding sequence, including L but without the initiation methionine, allowing the GFP to be separated from L. Sanger sequencing was used to sequence the full length of all mutant and WT viral clones (Biochemistry Sequencing Facility, University of Cambridge).

EMCV molecular clones were linearized with *BamH*I, and genomic RNA was *in vitro* transcribed using the T7 RiboMax kit (Promega). Transcripts were purified (RNeasy kit, Qiagen) before being transfected into BHK-21 cells to generate virus stocks.

### Preparation of virus stocks

BHK-21 cells were grown to 60%–70% confluency in 10 cm dishes. All Opti-MEM (Gibco) was supplemented with 1:1,000 RNaseOUT (Invitrogen). A volume of 90 µL Lipofectamine 2000 (Invitrogen) was added to 1.8 mL Opti-MEM, and 14 µg RNA was added to another 1.8 mL Opti-MEM. These solutions were mixed together and incubated for 20 min while the cells were washed with PBS. Following the addition of the transfection mixture, cells were incubated for 3.5 h at room temperature with gentle agitation. Transfection medium was removed prior to the addition of 8 mL of 2% FCS DMEM and incubation for 24 h or until full CPE was visible. The plates were then scraped, and samples were freeze/thawed three times before removing dead cells and debris through light clarification. The supernatant was then frozen at −70°C in aliquots. Virus concentration was estimated by plaque assay.

### Single-step growth curves

BHK-21 and MEF cells were infected in confluent 12-well plates (MOI 5 or 3, as indicated). At each time point, the medium was removed and clarified by centrifugation at 2,500 × *g* for 5 min before being frozen at −70°C. A volume of 200 µL of PBS was added to each well to enable the collection of the intracellular virus. Infected cells were freeze/thawed three times prior to clarification to remove cellular debris. Viral titer in each fraction was then estimated by plaque assay.

### Plaque assays

BSR cells were seeded at 35% confluency in 6-well plates 24 h prior to infection. The media were removed, and the cells were washed once with PBS before the addition of 1 mL serum-free DMEM containing the specified dilution of virus. The infection was incubated at room temperature with continuous rocking for 1 h before the inocula were removed, the cells were washed once with PBS, and 3 mL of DMEM (2% FCS) containing 1% low melting point agarose (ThermoFisher Scientific) was added to each well. Cells were incubated for 48 h before being fixed with 4% formaldehyde and stained with toluidine blue. Plaques were counted manually, and their sizes were quantified using ImageJ (73, 74).

### Immunoblots

During validation of 2B*KO EMCV, all samples to be analyzed by immunoblot were frozen in RIPA buffer (ThermoFisher Scientific) containing 1:10,000 Benzonase nuclease (Sigma) and protease and phosphatase inhibitors (Halt, ThermoFisher Scientific) (“complete RIPA”). All samples were boiled in SDS-based protein loading dye (Laemmli buffer) containing 10 mM DTT for 7 min prior to electrophoresis. Following resolution, proteins were transferred to 0.2 µm nitrocellulose membranes by semi-dry electrotransfer in a Transblot turbo transfer system using recommended standard settings (Bio-Rad). Membranes were blocked with 5% (wt/vol) non-fat milk powder in PBS for a minimum of 3 h with continuous rocking. Primary antibodies were diluted in blocking buffer prior to incubation with the membrane for at least 2 h. Membranes were washed three times with TBS-Tween 20 (0.1%) before being incubated with the relevant secondary antibody for 1 h with continuous rocking. Membranes were again washed three times with TBS-Tween 20 (0.1%) before being imaged with the Odyssey CLx imaging system (LI-COR). Where indicated, signal intensity was quantified using Image Studio Lite (LI-COR).

### Quantitative proteomics and TMT labeling of co-immunoprecipitation samples from infected BHK-21 cells

BHK-21 cells were mock infected or infected with HA-tagged WT and HA-tagged 2B*KO EMCV at an MOI of 5.0 for 10 hpi in four 10 cm dishes per sample, each in triplicate. Due to the number of dishes, samples were separated into two groups and infected 1 h apart to ensure the number of IP samples would be manageable. The HA-tagged 2B* was then purified using the Pierce Magnetic HA-Tag IP/Co-IP Kit with wash buffer supplemented with Halt protease and phosphatase inhibitors (ThermoFisher Scientific). During the final wash, 12.5% of each sample was retained in separate tubes for western blot analysis. Following the removal of the buffer for the third and final wash from the rest of the sample, the beads were snap frozen. Samples were eluted from the beads by boiling in 2× SDS loading buffer and prepared for downstream proteomic analysis by SP3 sample preparation (75). In brief, samples were reduced and alkylated, followed by precipitation onto magnetic beads by the addition of ethanol to 80% (vol/vol) final concentration. Samples were then washed three times in 90% (vol/vol) ethanol and then resuspended in 100 mM TEAB containing 20 ng/µL Trypsin Gold and digested overnight. Following digestion, samples were removed from the beads and labeled with Tandem Mass Tag reagents (ThermoFisher Scientific), before quenching with the addition of hydroxylamine. Samples were desalted by stage-tipping before analysis by LC-MS/MS on a Dionex 3000 coupled in line to a Q-Exactive-HF mass spectrometer using data-dependent acquisition. Analysis was performed using MaxQuant (76) using a fasta file containing Syrian Golden Hamster and EMCV protein sequences. Raw data and the .fasta files used have been uploaded to the PRIDE repository (PXD062303) (77).

Downstream data analysis of the MaxQuant proteinGroups output file was conducted using Perseus 2.0.11 (78). Reverse database hits, common contaminants (MaxQuant contaminant list), and proteins only identified by site were removed. Data were normalized for equal protein loading based on the median total reporter intensity and then subjected to a log_2_(*x*) transformation. Replicate samples were grouped, and rows with <3 valid values in at least one group were removed. Missing data were imputed from the normal distribution before Student’s *t*-tests. Putative interaction partners were defined as having both −log_10_(*P*-value) > 1.3 (i.e., *P*-value < 0.05) and log_2_(fold change) > 1 (i.e., fold > 2) in both the comparison of HA2B* EMCV against mock and HA2B* EMCV against HA2B*KO EMCV.

### Anti-FLAG immunoprecipitation

BHK-21 and BSR cells were co-transfected with both pCAGG-HA2B* and a cloned FLAG-tagged putative interaction partner (pCAGG-X) in 10 cm dishes. Twenty-four hours after transfection, the cells were washed in ice-cold PBS before being scraped and lysed in 750 µL of lysis buffer (25 mM Tris HCl [pH 7.4], 150 mM NaCl, 1 mM EDTA, 1% NP40, and 5% glycerol, pH 7.4) for 10 min at 4°C. The lysates were clarified, and 50 µL was retained (input). The FLAG-tagged bait protein and any interacting proteins were purified using anti-FLAG resin (Sigma). Briefly, lysates were incubated with the resin with continuous rotation for a minimum of 2 h at 4°C, then washed three times in fresh lysis buffer. Following the removal of the final wash, the resin was boiled for 5 min in 60 µL of 2× protein loading dye (Laemmli buffer) in the absence of DTT. The undissolved resin was removed by centrifugation, and DTT was added (final concentration 5 mM) prior to reheating the sample for a further 3 min. Proteins were resolved by polyacrylamide gel electrophoresis and imaged by immunoblot against the epitope tags.

### Confocal microscopy

BSR cells were seeded at 20% confluency onto glass coverslips in 24-well plates, 24 h prior to transfection (see above). Twenty-four hours after transfection, cells were washed in PBS and fixed with 4% paraformaldehyde for 20 min at room temperature. Cells were washed three times both prior to and immediately following permeabilization with 0.5% Triton X-100 for 10 min. To reduce nonspecific binding, cells were blocked with 10% BSA for a minimum of 2 h at room temperature with gentle agitation before the addition of the relevant primary antibodies. Antibodies against the HA epitope (C29F4, Cell Signaling Technologies) and the FLAG epitope (F1804, Sigma) were each diluted 1:1,000, and the coverslips were incubated at 4°C overnight with continuous gentle rocking. Samples were washed three times in PBS before the addition of fluorophore-labeled secondary antibodies diluted in 10% BSA (anti-rabbit Alexa-fluor 488 [Invitrogen]; anti-rat Alexa-fluor 594 [Abcam]). Samples were again incubated with continuous rocking at room temperature for 1 h. Finally, samples were washed three times, and the coverslips were mounted upon glass slides. DAPI was included in the mounting media (Prolong gold antifade with DAPI, ThermoFisher Scientific). Samples were imaged at 63× on a LSM 700 laser scanning confocal microscope.

### Phos-Tag SDS-PAGE

BSR cells were transfected as described above. At 24 hpt, cells were lysed in Tris-buffered saline containing 0.5% NP-40 (pH 8.0). Control samples were treated with lambda protein phosphatase (New England Biolabs) according to the manufacturer’s instructions. After boiling with Laemmli buffer, samples were electrophoresed on 15% bis-acrylamide (37.5:1) gels supplemented with Phos-Tag acrylamide (50 µM) (AAL-107, Wako Chemicals) and 100 µM MnCl_2_ in a standard SDS Tris-Glycine electrophoretic buffer (192 mM glycine, 25 mM Tris base, and 3.5 mM SDS). After transfer to nitrocellulose membranes, differentially phosphorylated protein species were detected by immunoblot probing for the HA tag.

### Live cell imaging

BHK-21 cells infected with GFP-tagged viruses were imaged at regular intervals using the Incucyte live cell analysis system and the Incucyte 2022B Rev2 GUI software (Sartorius). Three regions were imaged per well using a 10× objective (96-well plate, each sample in technical triplicate). The background threshold for each plate was independently set using manually selected images with high and low fluorescence levels. Cell confluence was measured by phase microscopy. GCU at each time point was normalized to the respective GCU in that well at 5.5 h post-infection.

### *In silico* protein structure prediction

Alphafold (79), I-TASSER (80), and Netsurfp 3.0 (81) were used to predict the secondary structure of the 2B* monomer, using the amino acid sequence from GenBank accession DQ294633.1. Structure of 2B* (GenBank DQ294633.1, unless otherwise stated) in complex with homodimer of 14-3-3ϵ (Uniprot P62258 residues 3-232) (82) was predicted using AlphaFold3 via the AlphaFold Server (36). Where indicated, serine residues were predicted as phosphoserine. Molecular graphics were generated using PyMOL (Schrödinger).

## Data Availability

Raw data and the .fasta files used have been uploaded to the PRIDE repository (PXD062303) (77).

## SUPPLEMENTAL MATERIAL

Figure S1 mbio.01008-25-s0001.pdf The 2B*KO mutation does not affect RNA replication in an EMCV replicon system.

Figure S2 mbio.01008-25-s0002.pdf SDS-PAGE and immunoblotting.

Figure S3 mbio.01008-25-s0003.pdf Overexpressed HA2B* and 14-3-3 isoforms all display cytosolic distribution.

Figure S4 mbio.01008-25-s0004.pdf Overexpressed HA2B* and 14-3-3 isoforms all display cytosolic distribution.

Figure S5 mbio.01008-25-s0005.pdf Overexpressed HA2B* and 14-3-3 isoforms all display cytosolic distribution.

Figure S6 mbio.01008-25-s0006.pdf Overexpressed ΔRRNSS HA2B* and 14-3-3 isoforms all display cytosolic distribution.

Figure S7 mbio.01008-25-s0007.pdf Reciprocal immunoprecipitation indicates that 2B* forms a ternary complex with a 14-3-3 dimer.

Figure S8 mbio.01008-25-s0008.pdf Predictions of 2B*:14-3-3 complex structures.

Figure S9 mbio.01008-25-s0009.pdf Overexpressed HA2B* is phosphorylated at serine 128 only.

Legends mbio.01008-25-s0010.docx Supplemental figure legends.

ASM does not own the copyrights to Supplemental Material that may be linked to, or accessed through, an article. The authors have granted ASM a non-exclusive, world-wide license to publish the Supplemental Material files. Please contact the corresponding author directly for reuse.
