# Supplementary figures and images for: Encephalomyocarditis virus protein 2B* interacts with 14-3-3 proteins through a phosphorylated C-terminal binding motif
Source: mBio. 2025 Aug 18;16(9):e01008-25. doi: 10.1128/mbio.01008-25 (PMC12421828; doi:10.1128/mbio.01008-25)

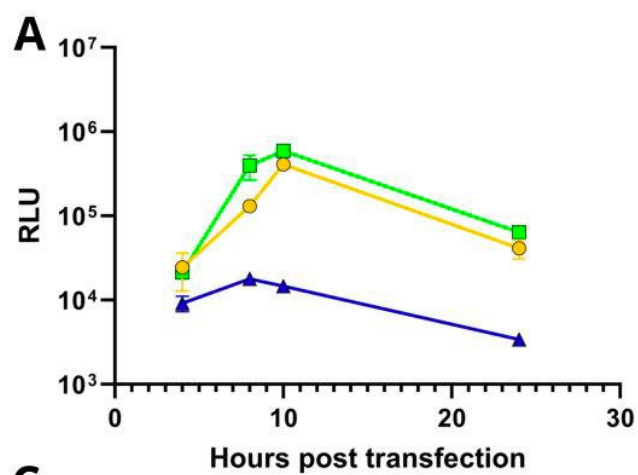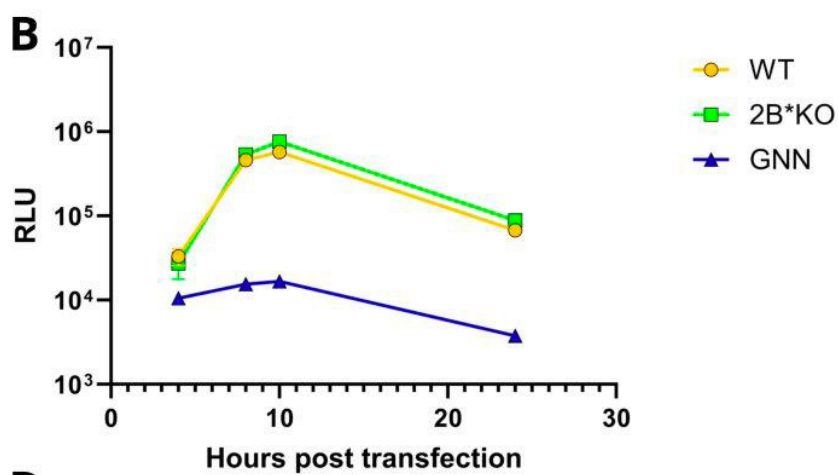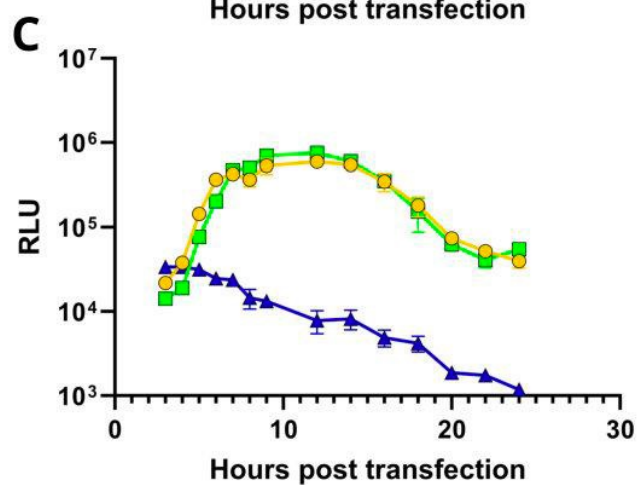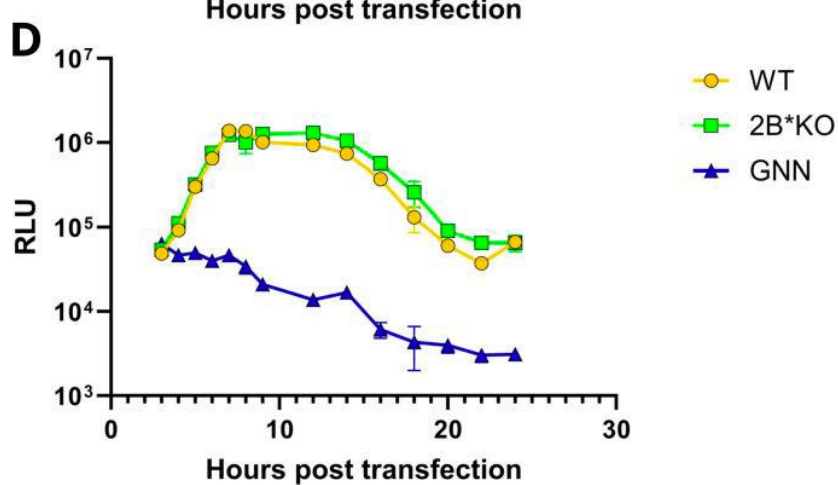

Supplement: Figure S1 — The 2B*KO mutation does not affect RNA replication in an EMCV replicon system. [file mbio.01008-25-s0001.pdf]

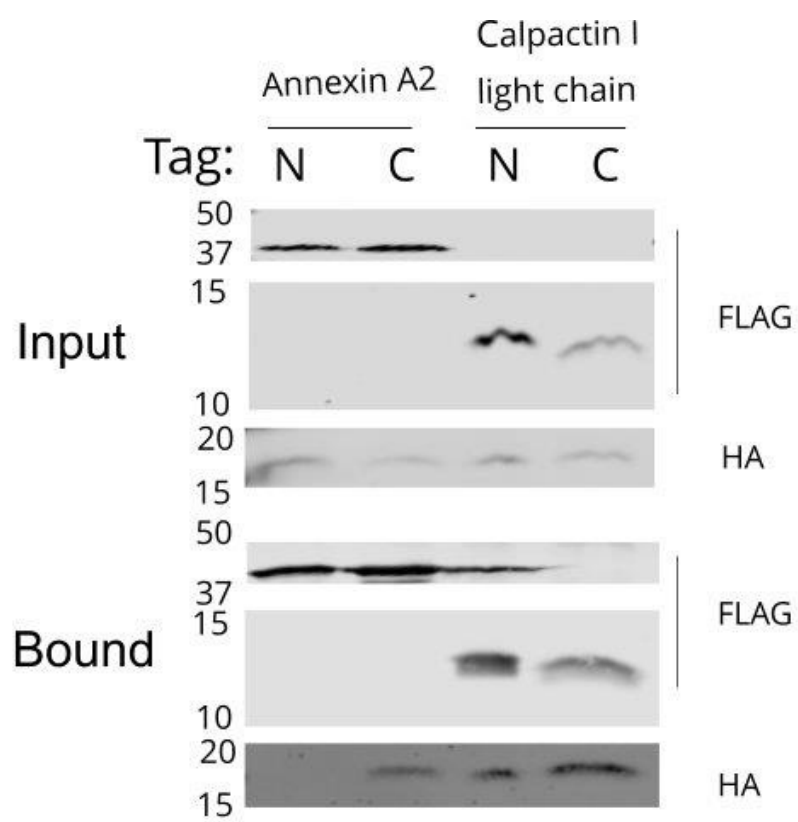

Supplement: Figure S2 — SDS-PAGE and immunoblotting. [file mbio.01008-25-s0002.pdf]

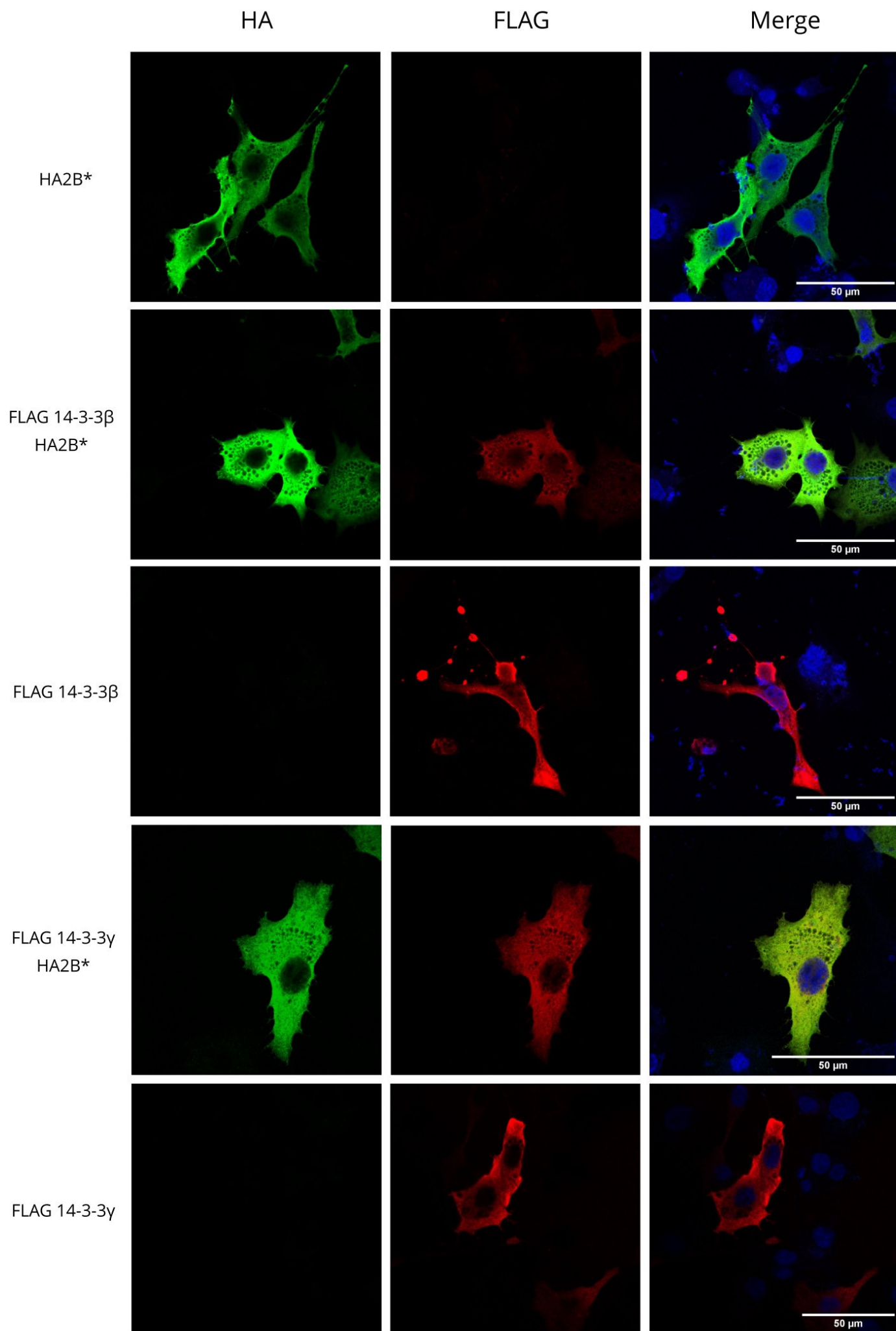

Supplement: Figure S3 — Overexpressed HA2B* and 14-3-3 isoforms all display cytosolic distribution. [file mbio.01008-25-s0003.pdf]

HA

FLAG

Merge

FLAG 14-3-3 $\epsilon$   
HA2B\*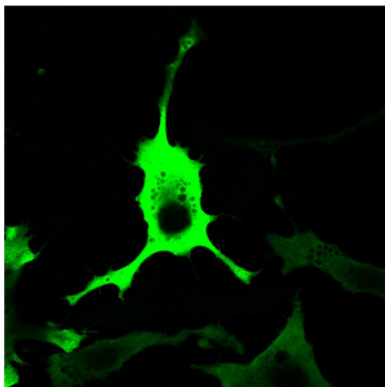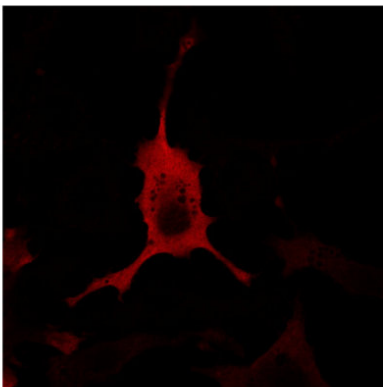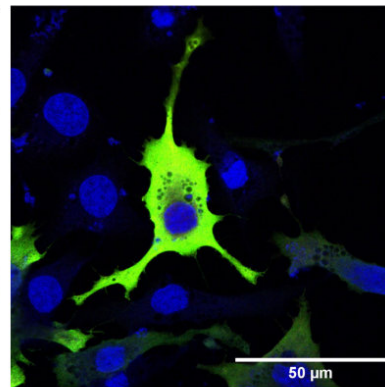FLAG 14-3-3 $\epsilon$ 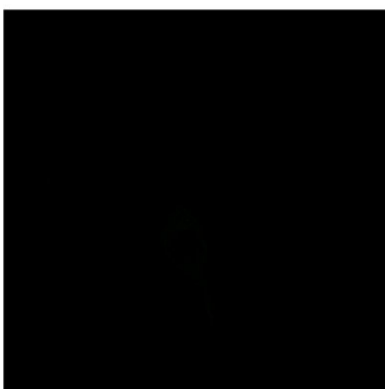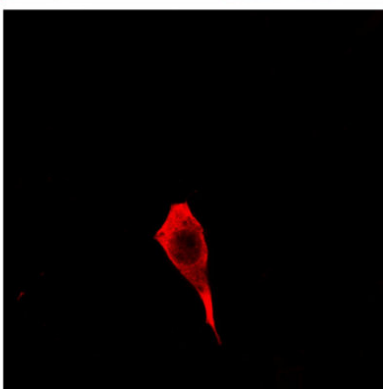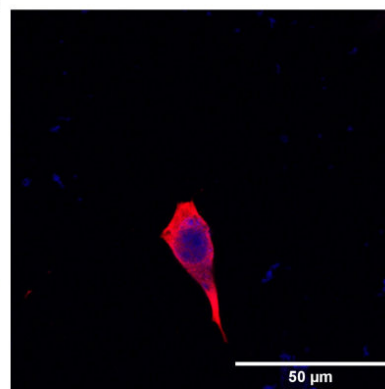FLAG 14-3-3 $\zeta$   
HA2B\*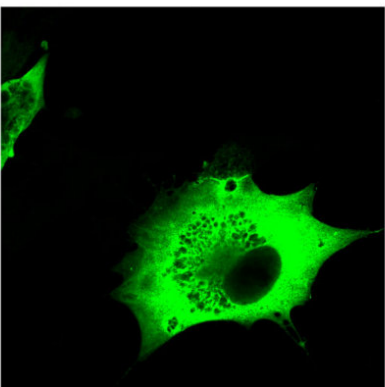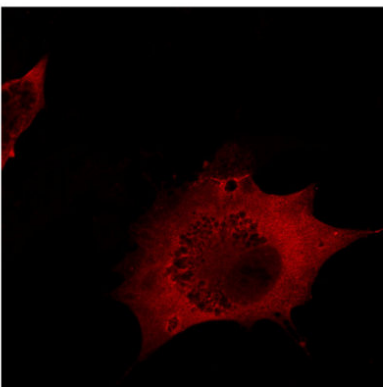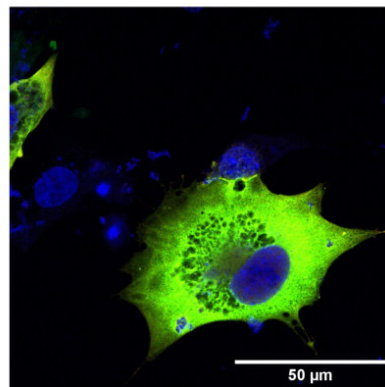FLAG 14-3-3 $\zeta$ 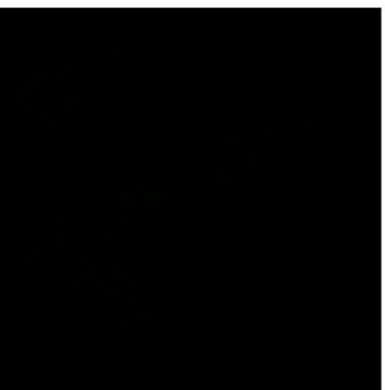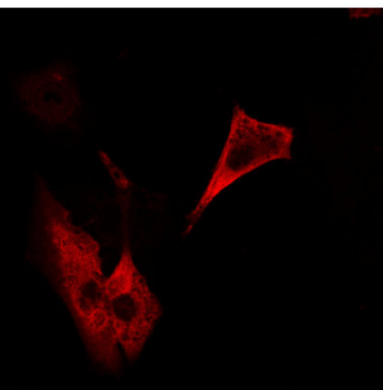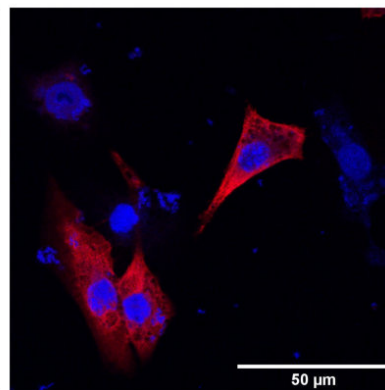FLAG 14-3-3 $\eta$   
HA2B\*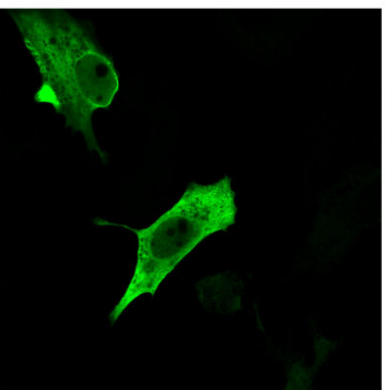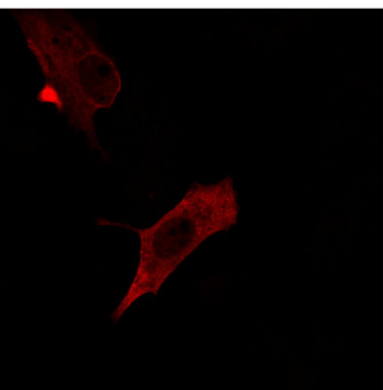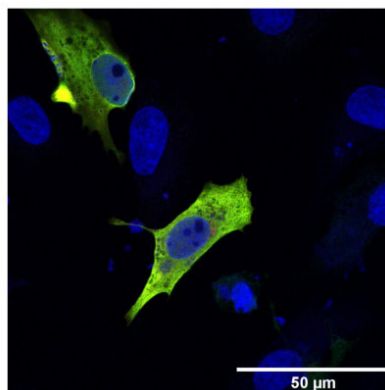

Supplement: Figure S4 — Overexpressed HA2B* and 14-3-3 isoforms all display cytosolic distribution. [file mbio.01008-25-s0004.pdf]

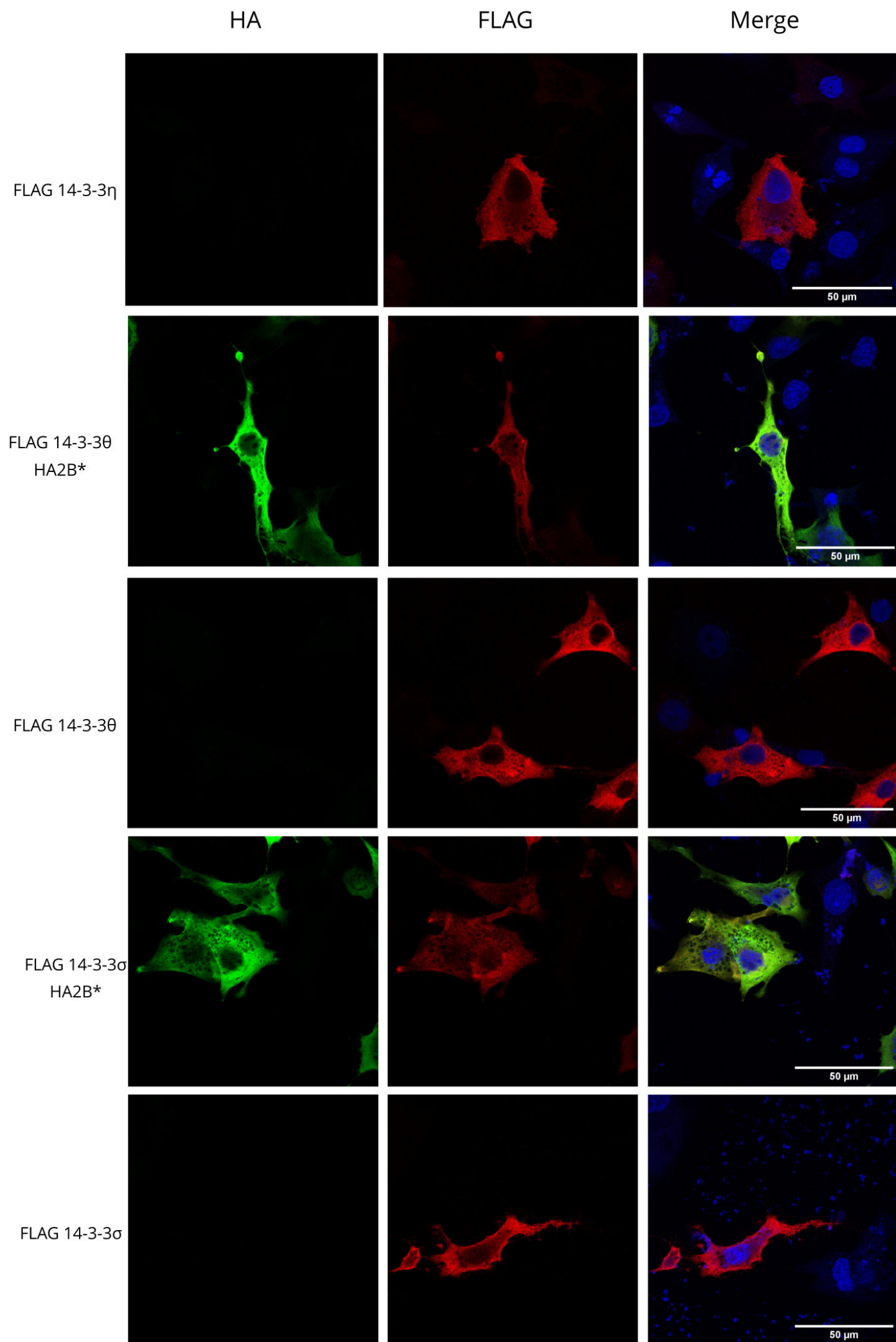

Supplement: Figure S5 — Overexpressed HA2B* and 14-3-3 isoforms all display cytosolic distribution. [file mbio.01008-25-s0005.pdf]

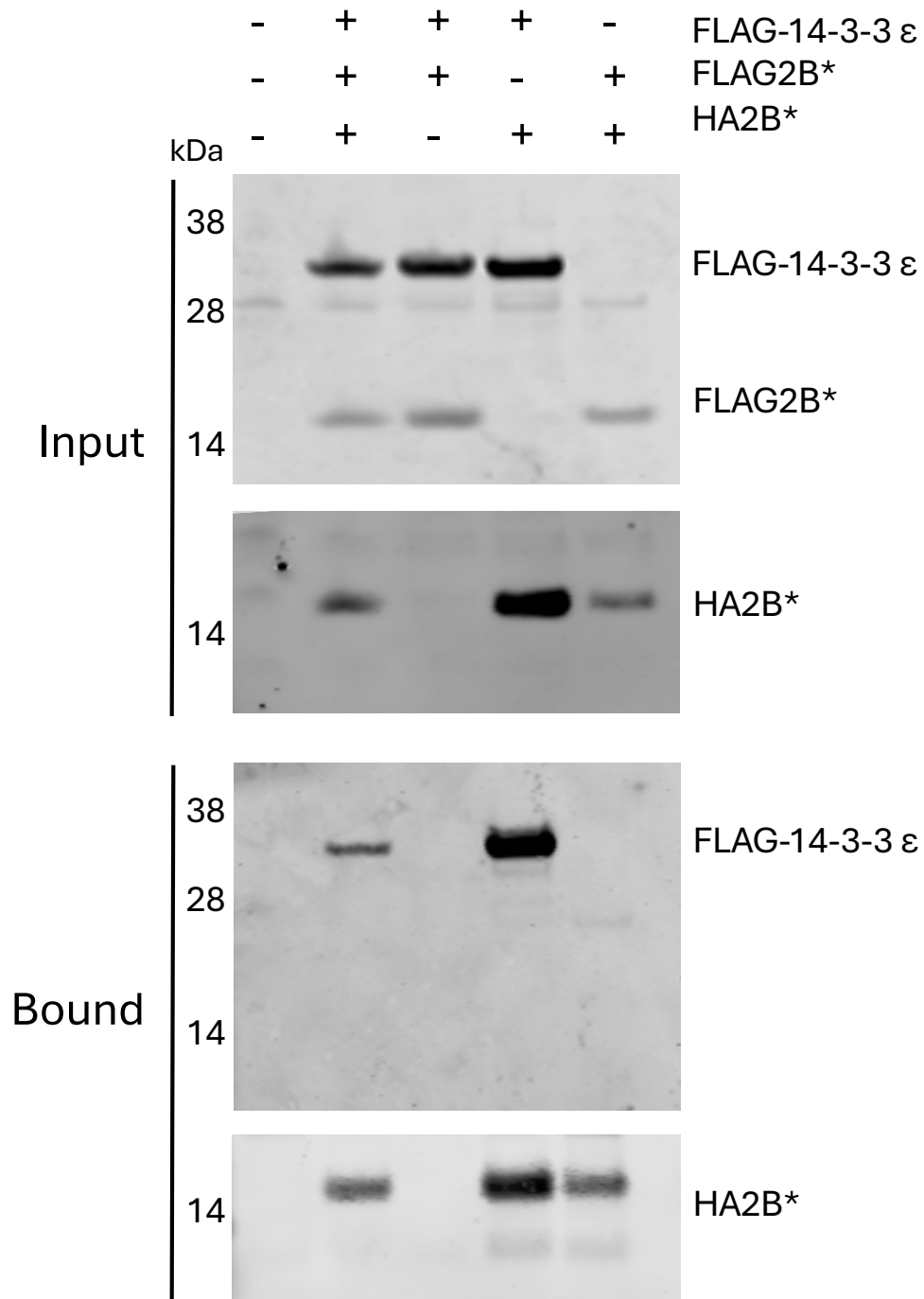

Supplement: Figure S7 — Reciprocal immunoprecipitation indicates that 2B* forms a ternary complex with a 14-3-3 dimer. [file mbio.01008-25-s0007.pdf]

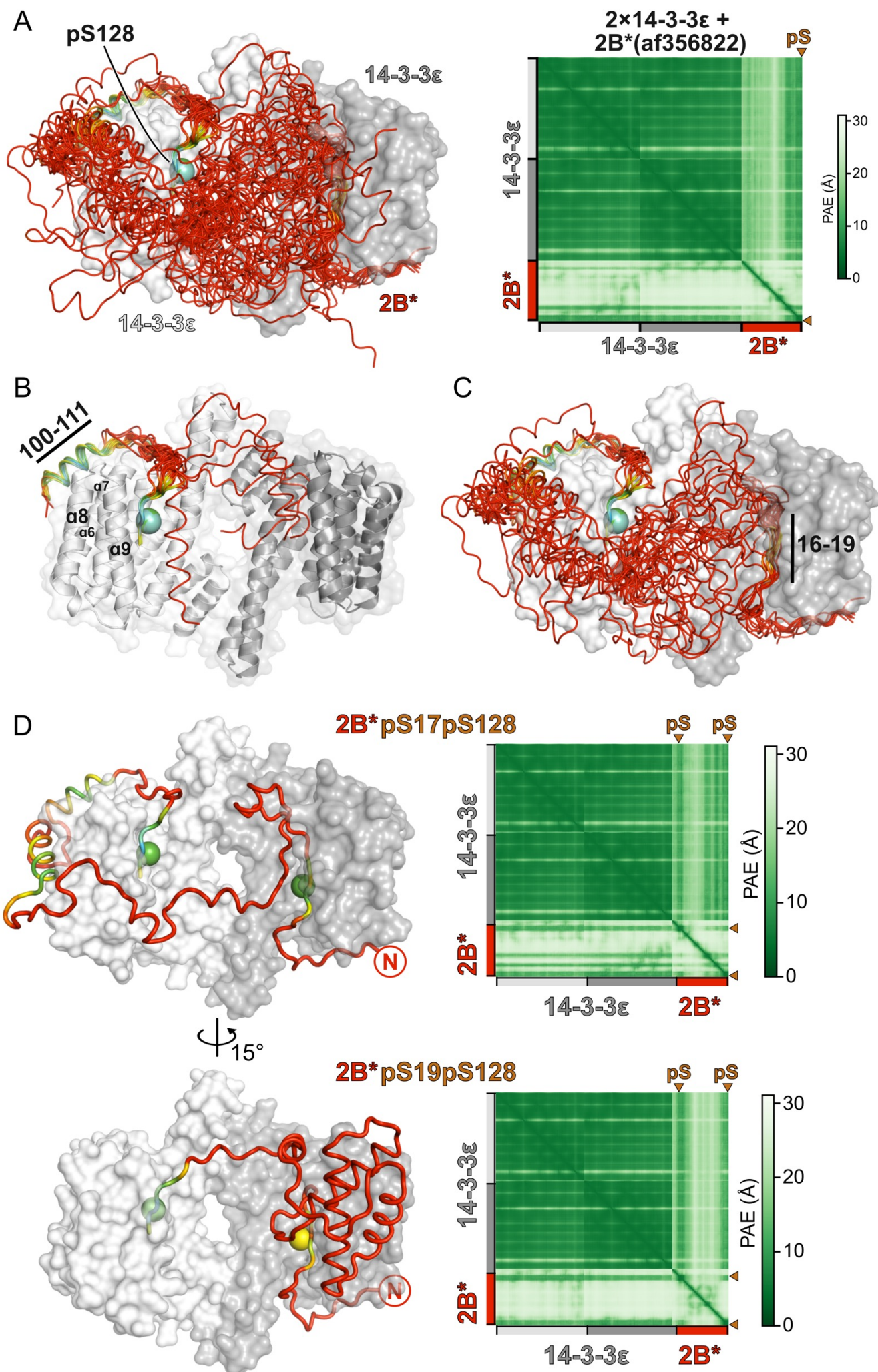

Supplement: Figure S8 — Predictions of 2B*:14-3-3 complex structures. [file mbio.01008-25-s0008.pdf]

**A**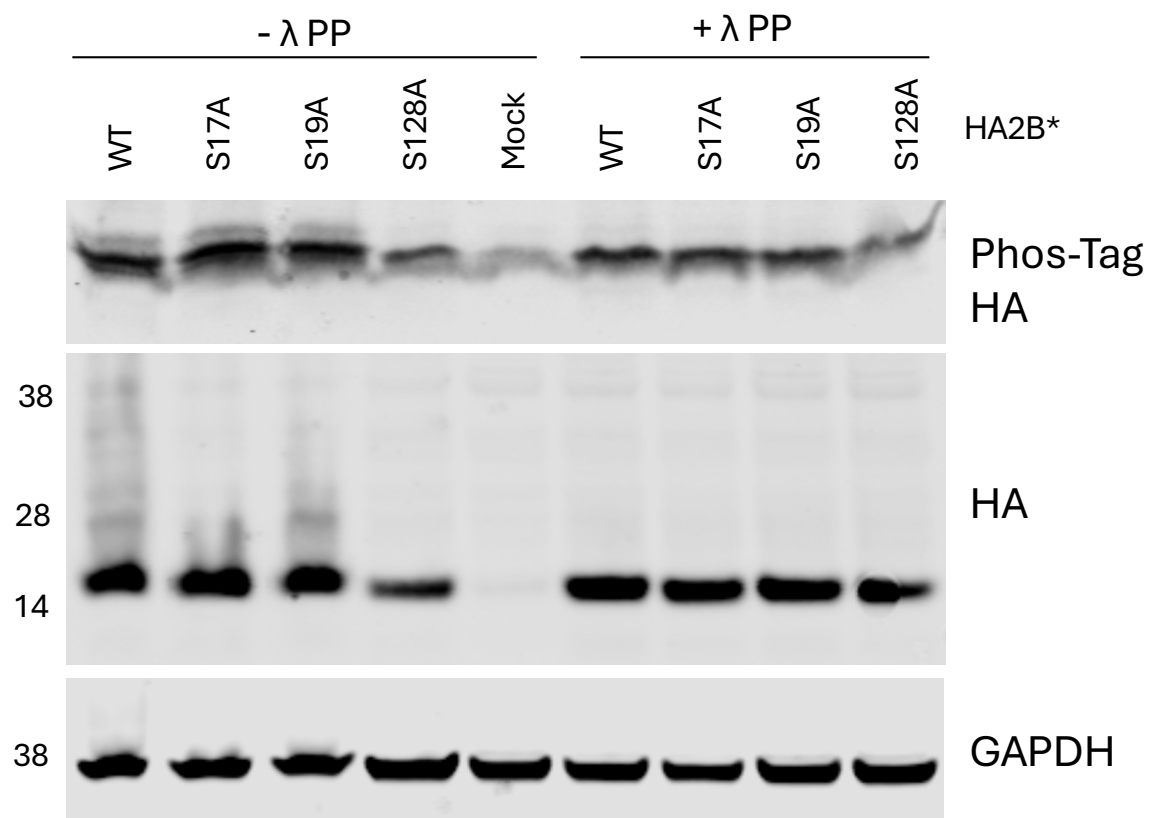**B**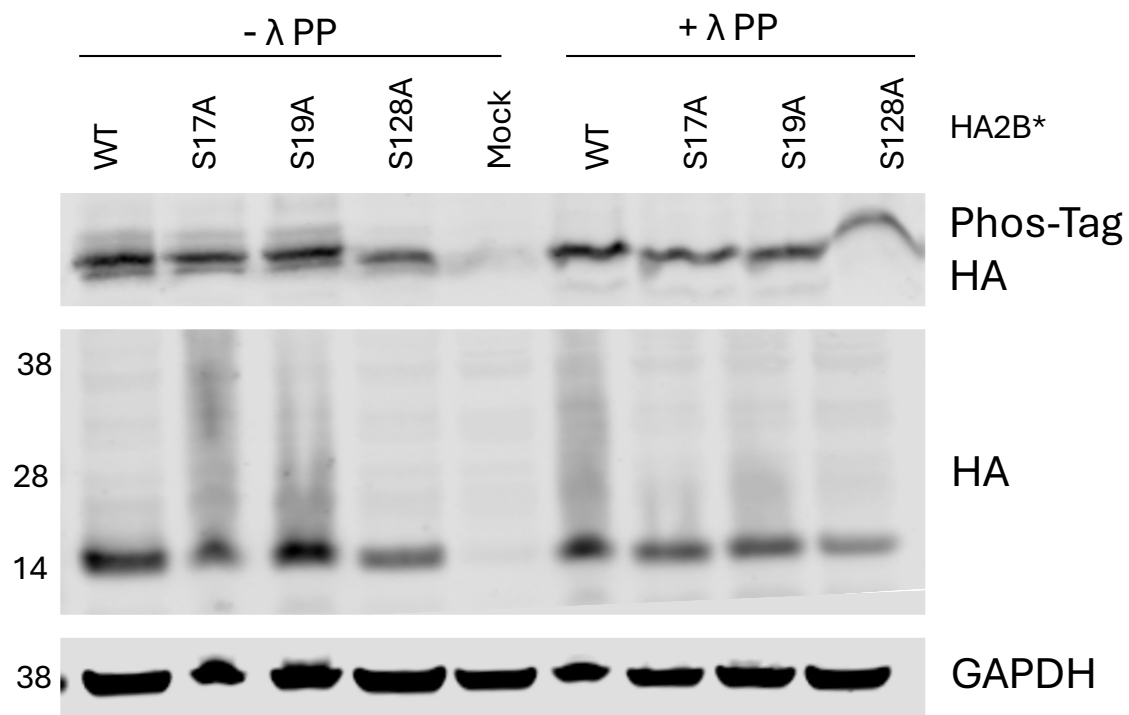

Supplement: Figure S9 — Overexpressed HA2B* is phosphorylated at serine 128 only. [file mbio.01008-25-s0009.pdf]
