# Supplementary material for: Encephalomyocarditis virus protein 2B* interacts with 14-3-3 proteins through a phosphorylated C-terminal binding motif
Source: mBio. 2025 Aug 18;16(9):e01008-25. doi: 10.1128/mbio.01008-25 (PMC12421828; doi:10.1128/mbio.01008-25)
Supplement: Figure S6 — Overexpressed ΔRRNSS HA2B* and 14-3-3 isoforms all display cytosolic distribution. [file mbio.01008-25-s0006.pdf]

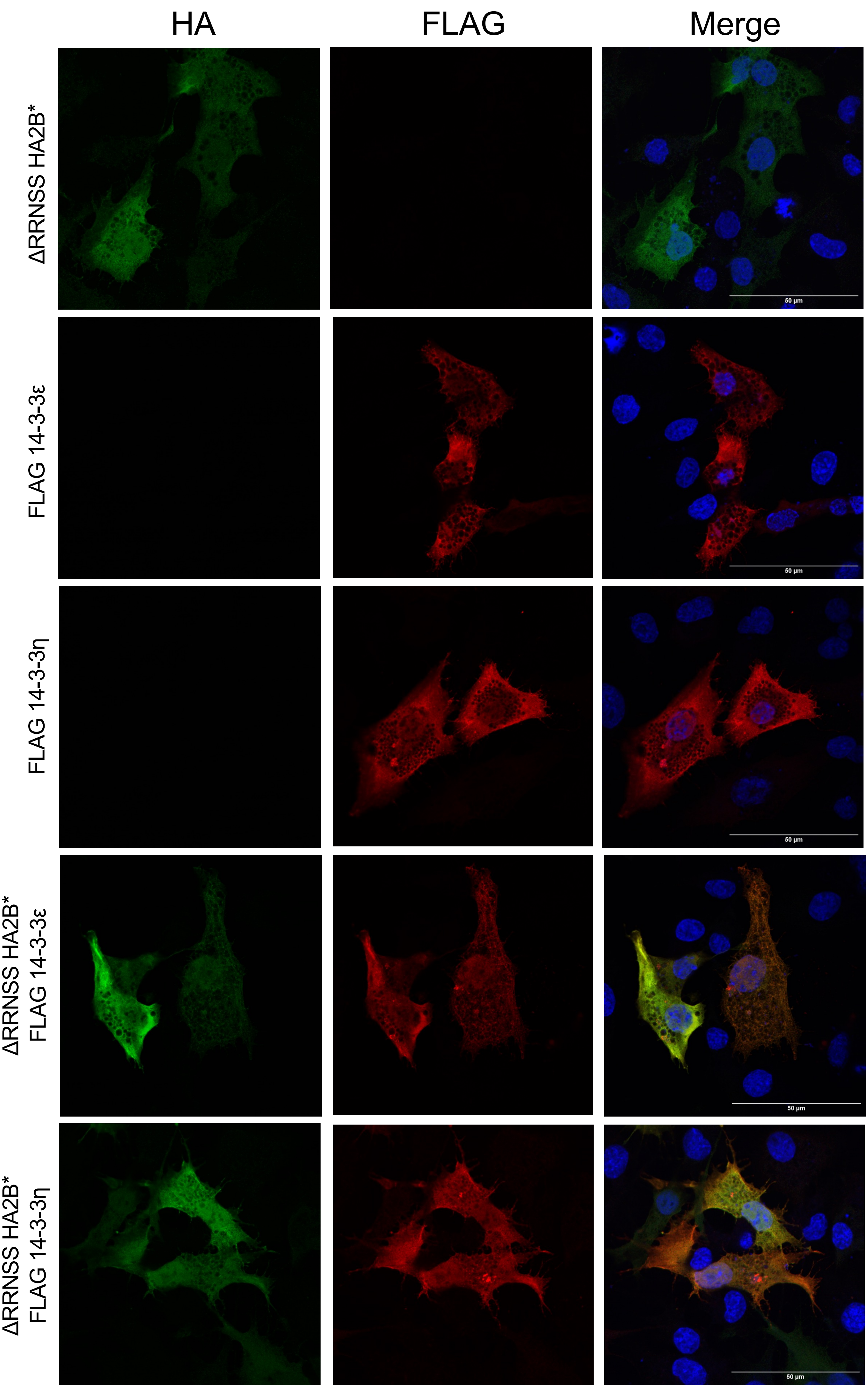
HA
FLAG
Merge
ΔRRNSS HA2B*
FLAG 14-3-3ε
FLAG 14-3-3η
ΔRRNSS HA2B* FLAG 14-3-3ε
ΔRRNSS HA2B* FLAG 14-3-3η
50 µm
