# Supplementary material for: Encephalomyocarditis virus protein 2B* interacts with 14-3-3 proteins through a phosphorylated C-terminal binding motif
Source: mBio. 2025 Aug 18;16(9):e01008-25. doi: 10.1128/mbio.01008-25 (PMC12421828; doi:10.1128/mbio.01008-25)
Supplement: Legends — Supplemental figure legends. [file mbio.01008-25-s0010.docx]

**Figure S1:** **The 2B*KO mutation does not affect RNA replication in an EMCV replicon system.** Various numbers of BHK-21 cells (3 x 10^4^ (A), 5 x 10^4^ (B), or 6.5 x 10^4^ (C, D)) were reverse transfected with various amounts of *in vitro* transcribed RNA (150 ng (A, B, C), or 260 ng (D)) from each EMCV replicon and seeded into a 96 well plate. At various time points from 3 h p.t., media was removed and replaced with PLB prior to freezing. Once all time points were collected, samples were thawed and firefly luciferase activity was measured. Error bars represent the standard deviation of triplicate wells. Only one biological repeat was carried out per condition.

**Figure S2:** A monolayer of BHK-21 cells was co-transfected with equal concentrations of pCAGG-HA2B* and the specified FLAG-tagged expression construct. After 24 h, cells were lysed and 10% of the lysate (input) was retained. The remainder was immunoprecipitated via the FLAG epitope. Samples were subjected to SDS-PAGE and immunoblotting using the indicated antibodies. Data shown are representative of two independent biological repeats. N, N-terminal FLAG tag; C, C terminal FLAG tag.

**Figure S3:** **Overexpressed HA2B* and 14-3-3 isoforms all display cytosolic distribution (1).** BSR cells were co-transfected with pCAGG-HA2B* and either the gene encoding 14-3-3β or 14-3-3γ, with an N-terminal FLAG tag, also in a pCAGG expression construct. Cells were fixed at 24 h and stained with both anti-HA and anti-FLAG antibodies. Nuclei were stained with DAPI during mounting. Images are representative of three independent biological repeats.

**Figure S4:** **Overexpressed HA2B* and 14-3-3 isoforms all display cytosolic distribution (2).** BSR cells were co-transfected with pCAGG-HA2B* and the gene encoding 14-3-3ε (isoform X1), 14-3-3ζ or 14-3-3η with an N-terminal FLAG tag, also in a pCAGG expression construct. Cells were fixed at 24 h and stained with both anti-HA and anti-FLAG antibodies. Nuclei were stained with DAPI during mounting. Images are representative of three independent biological repeats.

**Figure S5:** **Overexpressed HA2B* and 14-3-3 isoforms all display cytosolic distribution (3).** BSR cells were co-transfected with pCAGG-HA2B* and the gene encoding 14-3-3η, 14-3-3θ or 14-3-3σ, with an N-terminal FLAG tag, also in a pCAGG expression construct. Cells were fixed at 24 h and stained with both anti-HA and anti-FLAG antibodies. Nuclei were stained with DAPI during mounting. Images are representative of three independent biological repeats.

**Figure S6:** **Overexpressed** Δ**RRNSS HA2B* and 14-3-3 isoforms all display cytosolic distribution.** BSR cells were co-transfected with pCAGG-HAΔRRNSS 2B* and the gene encoding 14-3-3ε (isoform X1) or 14-3-3η, with an N-terminal FLAG tag, also in a pCAGG expression construct. Cells were fixed at 24 h and stained with both anti-HA and anti-FLAG antibodies. Nuclei were stained with DAPI during mounting. Images are representative of three independent biological repeats.

**Figure S7: Reciprocal immunoprecipitation indicates that 2B* forms a ternary complex with a 14-3-3 dimer.** BSR cells were co-transfected with various combinations of pCAGG-HA2B*, pCAGG-FLAG2B*, pCAGG-FLAG-14-3-3ε and empty vector. Cell lysates were immunoprecipitated with anti-HA antibody and subjected to SDS-PAGE and immunoblotting. Data shown are representative of three independent biological repeats.

**Figure S8: Predictions of 2B*:14-3-3 complex structures. (A)** AlphaFold3 predictions of 14-3-3ϵ homodimer in complex with 23 unique *Cardiovirus rueckerti* (Cardiovirus A) 2B* sequences (7) where the penultimate serine residue was modelled as phosphoserine. In all, this phosphoserine residue occupies the 14-3-3ϵ amphipathic groove. 14-3-3ϵ protomers are shown as semi-transparent molecular surfaces (grey) and 2B* is shown as a ribbon coloured by residue by prediction confidence (predicted local distance difference test; pLDDT), from red (low confidence, pLDDT ≤ 50) to blue (high confidence, pLDDT ≥ 90), with phosphate groups of phosphoserine shown as spheres. The predicted aligned error (PAE) of one representative model is shown (right). **(B)** Residues 100 onwards of all 2B* sequences are shown, highlighting the short helix (residues 100–111) that is predicted with moderate confidence to interact with helix α6 and the turn between helices α7 and α8 in 14-3-3ϵ for 18/23 2B* sequences. **(C)** Models where the 2B* motif containing conserved serine residues (residues 16–19) is predicted to interact with the opposing 14-3-3ϵ amphipathic groove. 2B* models shown correspond to GenBank sequences AF356822, DQ288856, DQ835185, FJ604852, KF293299, KF598860, KF598862, KF598864, KF836389, KX231802, MN547968, MT085334 and MT499387. **(D)** AlphaFold3 predictions of a 14-3-3ϵ homodimer in complex with 2B* where residues 17 and 128 (top) or 19 and 128 (bottom) are modelled as phosphoserines. 2B* is shown as a ribbon coloured by residue by pLDDT as in (A) and phosphate groups of phosphoserines are shown as spheres. The PAE of the complexes is shown (right), indicating areas of high confidence that coincide with the positions of the phosphoserine residues. Atomic coordinates and quality statistics for all models are presented in supplementary data file 1.

**Figure S9: Overexpressed HA2B* is phosphorylated at serine 128 only.** BSR cells were transfected with pCAGG-HA2B* or mutants thereof containing S17A, S19A or S128A. Lysates were harvested and either treated with lambda protein phosphatase (PP) or mock treated, prior to electrophoretic separation on a 15% acrylamide gel supplemented with 50 µM Phos-Tag^TM^ acrylamide (upper panel). Cell lysates were also subjected to standard SDS-PAGE and immunoblotting (centre and lower panels). Two of the three experimental repeats are shown here, with the third repeat shown in Figure 5F.
